# Real-world characteristics, testing patterns, and outcomes in patients with tropomyosin receptor kinase fusion cancer from a large pooled clinicogenomic database

**DOI:** 10.1016/j.esmorw.2026.100814

**Published:** 2026-11-05

**Authors:** C.B. Westphalen, M.S. Brose, S. Bhattacharjee, V. Bernard-Gauthier, X. Pan, M. Kurtinecz, H. Guo, V. Aris, H. Seidel, J. Zong, D.-I. Burcoveanu, V. Subbiah, K.L. Kehl, A. Drilon

**Affiliations:** 1 Comprehensive Cancer Center (CCC Munich LMU), LMU University Hospital, LMU Medizin, Ludwig-Maximilians-Universität München, Munich, Germany; 2 Department of Medicine III, LMU University Hospital, LMU Medizin, Ludwig-Maximilians-Universität München, Munich, Germany; 3 German Cancer Consortium (DKTK), Partner Site Munich, Munich, Germany; 4 Sidney Kimmel Comprehensive Cancer Center, Department of Medical Oncology, Thomas Jefferson University, Philadelphia, USA; 5 Real World Evidence Center of Excellence, Bayer HealthCare Pharmaceuticals, Inc., Whippany, USA; 6 Global Medical & Evidence, Bayer Pharmaceuticals, Inc., Toronto, Canada; 7 Global Medical & Evidence, Oncology, Bayer HealthCare Pharmaceuticals, Inc., Whippany, USA; 8 Data Sciences, Bayer HealthCare Pharmaceuticals, Inc., Whippany, USA; 9 Digital Health & Real World Science, Bayer Pharmaceuticals, Inc., Mississauga, Canada; 10 Digital Health & Real World Science, Bayer HealthCare Pharmaceuticals, Inc., Whippany, USA; 11 Translational Sciences Oncology/Computational Biology, Bayer AG, Berlin, Germany; 12 Clinical Development of Oncology, Bayer HealthCare Pharmaceuticals, Inc., Basel, Switzerland; 13 Division of Oncology, Department of Medicine, Stanford Cancer Institute, Stanford University School of Medicine, Stanford, USA; 14 Department of Medical Oncology, Dana-Farber Cancer Institute, Boston, USA; 15 Department of Medicine, Harvard Medical School, Boston, USA; 16 Thoracic Oncology Service, Memorial Sloan Kettering Cancer Center, New York, USA; 17 Department of Medicine, Weill Cornell Medical College, New York, USA

**Keywords:** biomarker testing patterns, clinical outcomes, *NTRK* gene fusion, real-world evidence, treatment patterns, TRK fusion cancer

## Abstract

**Background:**

Tropomyosin receptor kinase (TRK) inhibitors are highly active in neurotrophic tyrosine receptor kinase (*NTRK*) gene fusion cancers. The registrational datasets of the three approved TRK inhibitors are well described in the literature but real-world data remain limited. To address this gap, we conducted the Real wOrld treAtment anD outcoMes Among Patients with TRK fusion cancer study.

**Patients and methods:**

This pooled analysis of five large, international clinicogenomic databases assessed patient characteristics, biomarker testing patterns, co-occurring genomic alterations, and clinical outcomes of patients with TRK fusion cancers.

**Results:**

A total of 451 patients with advanced/metastatic TRK fusion cancer were identified. *NTRK* gene fusions were mainly identified by next-generation sequencing [363 (81%) patients]. The median time from diagnosis of advanced/metastatic cancer to the date of the first *NTRK* gene fusion test was 25 days [interquartile range (IQR) 0-459]. Of the 172 (38%) patients who received a TRK inhibitor, the median time from *NTRK* gene fusion testing to first TRK inhibitor administration was 118 days (IQR 31-335). The median real-world overall survival was 28 months [95% confidence interval (CI) 21-32] for any first-line systemic therapy and it varied widely across tumor types, from 11 months (95% CI 8-17) for pancreatic cancer to 41 months (95% CI 25-50) for breast cancer.

**Conclusions:**

This is the largest integrated real-world cohort describing patients with TRK fusion cancer to date, providing a comprehensive picture of TRK fusion cancer treatment in the age of TRK-targeted therapy.

## Highlights

• *NTRK* gene fusions were identified by NGS in 81% of patients (363 of 451).
• Median time from advanced cancer diagnosis to *NTRK* testing: 25 days (IQR 0-459).
• Median time from *NTRK* testing to first TRK inhibitor: 118 days (IQR 31-335).
• Median rwOS for any first-line systemic therapy: 28 months (95% CI 21-32).
• rwOS ranged from 11 months (pancreatic cancer) to 41 months (breast cancer).

## Introduction

Neurotrophic tyrosine receptor kinase (*NTRK*) gene fusions are found in <1% of solid tumors. Up to 90% of certain rare cancers (e.g. secretory carcinoma of the salivary gland and infantile fibrosarcoma) and <0.5% of more common cancers [e.g. colorectal cancer (CRC) and non-small-cell lung cancer (NSCLC)] harbor these fusions.^1^^,^^2^ Tropomyosin receptor kinase (TRK) fusion proteins are actionable therapeutic targets. Two first-generation TRK inhibitors (larotrectinib and entrectinib) are currently approved for the treatment of TRK fusion cancers, irrespective of tumor type and patient age.^3^, ^4^, ^5^, ^6^, ^7^ In clinical trials, larotrectinib had an overall response rate (ORR) of 65% [95% confidence interval (CI) 59-70]^8^^,^^9^ and entrectinib had an ORR of 62% (95% CI 55-69).^10^ A next-generation TRK inhibitor (repotrectinib) was approved for the treatment of TRK fusion cancers in June 2024.^11^

To date, few studies have examined the clinical characteristics and outcomes of patients with TRK fusion cancers. Studies evaluating the clinical efficacy of TRK inhibitors in the real-world setting are also limited. In a recent study of 164 patients with various TRK fusion cancers evaluating the real-world efficacy of larotrectinib versus standard-of-care, treatment with larotrectinib was associated with longer overall survival (median not reached versus 37.2 months; HR 0.44, 95% CI 0.23-0.83).^12^ Furthermore, published real-world studies are limited by small sample sizes and a lack of generalizability.^12^, ^13^, ^14^, ^15^, ^16^, ^17^, ^18^ These gaps stem from the rarity of TRK fusion cancers, the recent introduction of TRK inhibitors, and the limited data on testing patterns, treatment approaches, and clinical outcomes.

To address these gaps, we conducted the Real wOrld treAtment anD outcoMes Among Patients with TRK fusion cancer (ROADMAP) study, which aimed to provide further insights into TRK fusion cancer in the real-world setting through the analysis of large, pooled international clinicogenomic databases. The main objectives of this study were to describe the demographic and clinical characteristics of patients with advanced TRK fusion cancer, as well as biomarker testing patterns, treatment patterns, and clinical outcomes.

## Methods

### Study design

ROADMAP was a retrospective, observational, international cohort study (Figure 1). Reporting of this real-world study conforms to ESMO GROW guidelines (see Supplementary Table S1, available at https://doi.org/10.1016/j.esmorw.2026.100814).

**Figure 1:**
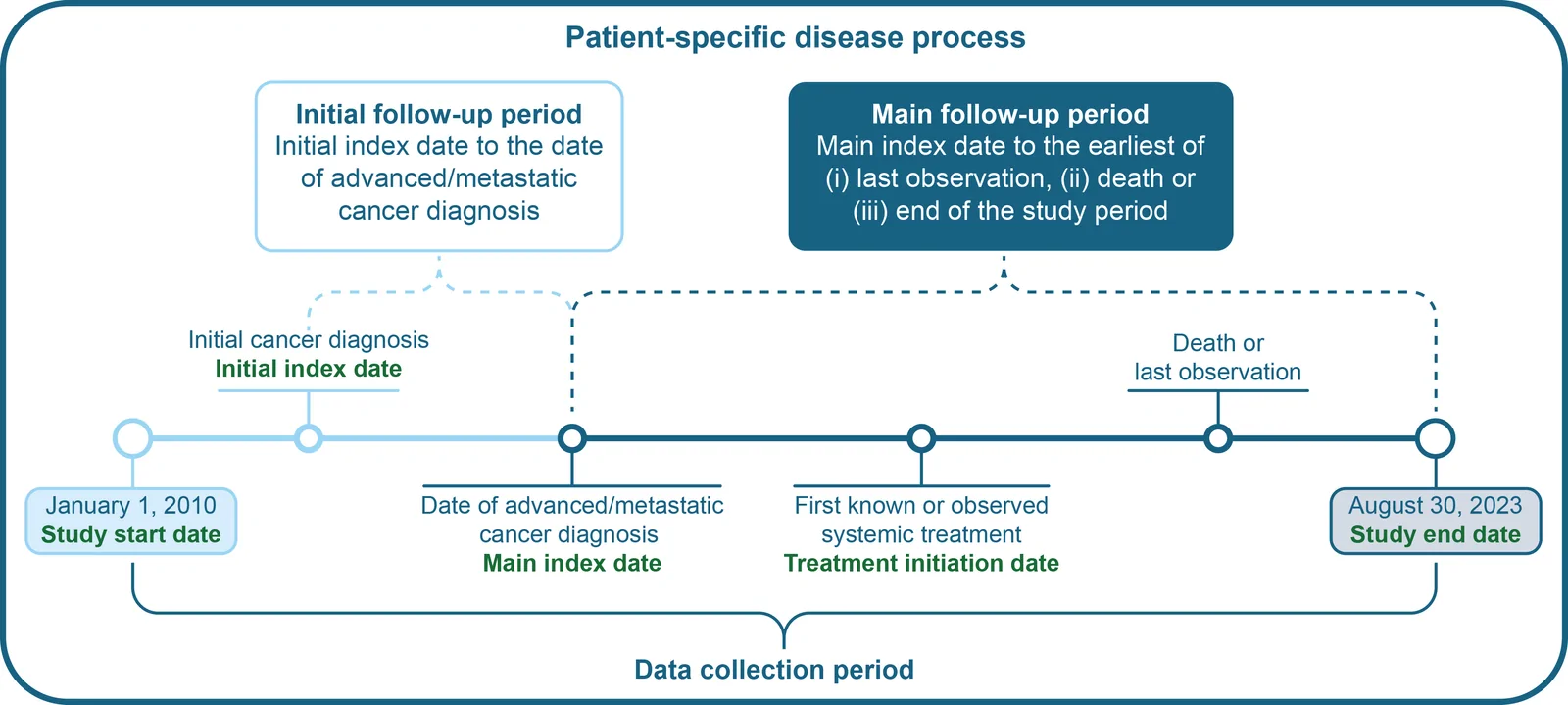
**Study design**.

### Study population

Adult (≥18 years) and pediatric (<18 years) patients with a diagnosis of advanced/metastatic TRK fusion cancer were included in the study. Tumor presence of an *NTRK* gene fusion was confirmed with next-generation sequencing (NGS), reverse transcriptase polymerase chain reaction, PCR, or FISH. Patients involved in any of the three larotrectinib clinical trials [NCT02122913, SCOUT (NCT02637687), and NAVIGATE (NCT02576431)] were excluded.

### Data sources

Data were pooled from five retrospective sources: a chart review of the American Association for Cancer Research Genomics Evidence Neoplasia Information Exchange (AACR-GENIE) international registry^19^; a chart review of the US-based Cardinal Health database^20^; deidentified electronic health records of the US-based Flatiron Health-Foundation Medicine Clinico-Genomic Database (FH-FMI CGDB)^21^; the US-based Oncology Research Information Exchange Network (ORIEN) data source^22^; and a global chart review of multiple sites across several countries (more information is available in Supplementary Methods, Data Sources, available at https://doi.org/10.1016/j.esmorw.2026.100814). Following pooling, data were deduplicated using a prespecified algorithm (see Supplementary Methods, Analyses, available at https://doi.org/10.1016/j.esmorw.2026.100814).

### Study timeframe

Data covered the period from 1 January 2010 to 30 August 2023 (Figure 1). As the approval of repotrectinib for the treatment of TRK fusion cancers in June 2024^11^ fell outside of this period, its utilization was not assessed in this study. The main index date was the date of diagnosis of advanced/metastatic cancer. The treatment initiation date was the date of the first known/observed systemic treatment following the main index date. The baseline period began on or up to 3 months (90 days) before the main index date. The follow-up period was the time from the main index date to the date of last observation, the end of the study period, or death, whichever occurred first.

### Variables assessed

This study examined the demographic and clinical characteristics during the baseline period. Biomarker testing patterns, treatment patterns, and clinical outcomes were also assessed.

### Clinical outcomes definitions

Line of therapy (LOT) was defined as a distinct treatment combination of systemic therapy, which included chemotherapy, immunotherapy, and targeted therapeutics. Retreatment with the same regimen was considered the same LOT. A component of the original combination could be dropped without incrementing the LOT; however, the LOT was incremented if new drugs were added to the original treatment regimen. The time from diagnosis of advanced/metastatic disease to the first *NTRK* gene fusion test result and the time from first *NTRK* gene fusion test result to initiation of the first TRK-targeted therapy treatment (larotrectinib or entrectinib) were recorded.

Clinical outcomes included real-world overall survival (rwOS), duration of therapy (DOT), time to next treatment (TTNT), real-world progression-free survival (rwPFS), and real-world response rate (rwRR). Of note, rwOS was defined as the time from initiation of each LOT to death. Patients were censored at the latest of either the date of the last structured recorded clinical activity or the end of the follow-up period for patients without a death event (Supplementary Table S2, available at https://doi.org/10.1016/j.esmorw.2026.100814). The time-to-event endpoint definitions used conform to previously published consensus recommendations.^23^

DOT was defined as the time from initiation of a LOT until permanent treatment discontinuation [defined as the end of a LOT followed by another LOT, therapy discontinued with physician’s note, discontinued for a predefined gap period (e.g. >90 days), or death for any reason]. For patients who discontinued a therapy of interest, DOT was measured as the time from the date of LOT initiation to the date of discontinuation. Patients who were still receiving a therapy of interest at the end of follow-up were censored at the date of last known activity for the specific LOT (defined as the last administration or noncancelled order of a drug contained within the treatment regimen).

TTNT was defined as the time from the first LOT initiation until the date of initiation of the next LOT or death for patients who received a subsequent LOT. Patients who did not receive a subsequent treatment (continuing current treatment at the time of the analysis or loss of follow-up not due to confirmed death) were censored at the date of last known activity (defined as the last administration or noncancelled order of a drug contained within the treatment regimen). For patients who did not receive a subsequent treatment and had a confirmed death, TTNT was measured as time from first LOT to date of death.

rwPFS was defined as the time from each LOT initiation to the date of progression or death (death must have occurred within 30 days after the last assessment or within 30 days after the first LOT end date). Patients whose disease had not progressed at the time of the analysis were censored using the date of the last valid disease assessment before data cut-off. For patients who initiated additional LOTs, censoring occurred at the last disease assessment before the initiation date of the subsequent LOT.

rwRR was defined as the proportion of patients with an assessment of real-world complete response or partial response after initiation of line-specific treatment among all patients.

Definitions and periods of assessment for each outcome variable evaluated are detailed in Supplementary Table S2, available at https://doi.org/10.1016/j.esmorw.2026.100814.

### Statistical analyses

#### Main analysis

Patients included in the main analysis were diagnosed with advanced/metastatic TRK fusion cancers (the advanced/metastatic cancer analysis set). All analyses were stratified by tumor subtype (breast, CRC, NSCLC, pancreatic, salivary gland, soft-tissue sarcoma, thyroid, and other cancers).

Data were summarized with descriptive statistics; no formal statistical hypotheses were tested. Categorical variables were presented as frequencies (counts and percentages). Continuous variables were summarized by means, medians, standard deviation, minimum, maximum, and interquartile range (IQR). A competing risk analysis of time from *NTRK* gene fusion test to TRK-targeted treatment was conducted using the cumulative incidence function method with death as a competing risk.

The frequency of missing values was reported in descriptive tables. rwOS, DOT, TTNT, and rwPFS were estimated using the Kaplan–Meier method and described using medians and 95% CIs and/or IQRs. In addition, the left truncation approach was applied to the rwOS analysis to reflect that patients only entered the risk set and began follow-up at the time of genomic sequencing. Analyses of rwOS, DOT, TTNT, and rwPFS were also stratified by LOT. rwRR was described using point estimates and 95% CI.

#### Exploratory analysis of all patients with TRK fusion cancers

For the exploratory endpoint, a wider cohort including all patients with a diagnosis of TRK fusion cancer during the study period, regardless of disease stage, was identified from the pooled data sources. Statistical methods and results of this exploratory analysis are presented in the Supplementary Materials, available at https://doi.org/10.1016/j.esmorw.2026.100814.

## Results

### Patient characteristics

A total of 451 patients (436 adult and 15 pediatric) with advanced/metastatic TRK fusion solid tumors were eligible for inclusion (Table 1 and Supplementary Figure S1, available at https://doi.org/10.1016/j.esmorw.2026.100814). The median age was 60 (range 0-85) years and 232 patients (51%) were female. The tumor types included were NSCLC (*n =* 70), CRC (*n =* 61), thyroid cancer (*n =* 47), soft-tissue sarcoma (*n =* 46), breast cancer (*n =* 44), salivary gland cancer (*n =* 32), pancreatic cancer (*n =* 32), and other cancers (*n =* 119; Table 1). All databases except the global chart review included only patients from the United States (see Supplementary Methods for data sources, available at https://doi.org/10.1016/j.esmorw.2026.100814); other countries included in the global chart review were five patients from Australia, three each from Canada and the United Kingdom, two from Italy and one from Taiwan. Baseline demographics and clinical characteristics by tumor type are shown in Table 1 and by age group in Supplementary Table S3, available at https://doi.org/10.1016/j.esmorw.2026.100814.

**Table 1:** Baseline demographics and clinical characteristics in patients with advanced/metastatic cancers by tumor type

Characteristics	NSCLC (*n =* 70)	CRC (*n =* 61)	Thyroid cancer (*n =* 47)	Soft-tissue sarcoma (*n =* 46)	Breast cancer (*n =* 44)	Salivary gland cancer (*n =* 32)	Pancreatic cancer (*n =* 32)	Other (*n=* 119)^a^	Total (*N =* 451)
Sex, *n* (%)									
Female	31 (44)	38 (62)	30 (64)	22 (48)	42 (96)	8 (25)	13 (41)	48 (40)	232 (51)
Male	39 (56)	23 (38)	17 (36)	23 (50)	2 (5)	24 (75)	19 (59)	71 (60)	218 (48)
Missing	0	0	0	1 (2)	0	0	0	0	1 (<1)
Age at advanced/metastatic diagnosis, years									
Median (range)	63 (22-84)	62 (45-84)	51 (7-82)	54 (0-85)	50 (9-81)	60 (39-79)	62 (39-85)	62 (0-83)	60 (0-85)
Age group at advanced/metastatic diagnosis, *n* (%)									
Age ≥18 years	70 (100)	61 (100)	42 (89)	41 (89)	43 (98)	32 (100)	32 (100)	115 (97)	436 (97)
Age <18 years	0	0	5 (11)	5 (11)	1 (2)	0	0	4 (3)	15 (3)
Race, *n* (%)									
White	58 (83)	40 (66)	32 (68)	32 (70)	31 (71)	26 (81)	27 (84)	95 (80)	341 (76)
Asian	7 (10)	3 (5)	6 (13)	4 (9)	1 (2)	2 (6)	2 (6)	7 (6)	32 (7)
Black	4 (6)	5 (8)	3 (6)	3 (7)	7 (16)	1 (3)	3 (9)	4 (3)	30 (7)
Other	0	7 (12)	3 (6)	6 (13)	2 (5)	3 (9)	0	7 (6)	28 (6)
Unknown/missing	1 (1)	6 (10)	3 (6)	1 (2)	3 (7)	0	0	6 (5)	20 (4)
Ethnicity, *n* (%)									
Hispanic or Latino	2 (3)	5 (8)	5 (11)	5 (11)	4 (9)	3 (9)	6 (19)	5 (4)	35 (8)
Not Hispanic or Latino	56 (80)	29 (48)	34 (72)	26 (57)	30 (68)	25 (78)	20 (63)	88 (74)	308 (68)
Unknown/missing	12 (17)	27 (44)	8 (17)	15 (33)	10 (23)	4 (13)	6 (19)	26 (22)	108 (24)
Region, *n* (%)									
South	12 (17)	3 (5)	0	2 (4)	2 (5)	1 (3)	7 (22)	14 (12)	41 (9)
West	7 (10)	3 (5)	4 (9)	3 (7)	0	5 (16)	3 (9)	9 (8)	34 (8)
Northeast	3 (4)	3 (5)	0	2 (4)	0	3 (9)	2 (6)	11 (9)	24 (5)
Midwest	3 (4)	2 (3)	2 (4)	1 (2)	1 (2)	1 (3)	0	1 (<1)	11 (2)
Unknown/missing	45 (64)	50 (82)	41 (87)	38 (83)	41 (93)	22 (69)	20 (63)	84 (71)	341 (76)
Smoking status, *n* (%)									
History of smoking	37 (53)	3 (5)	4 (9)	6 (13)	1 (2)	4 (13)	10 (31)	16 (13)	81 (18)
No history of smoking	20 (29)	5 (8)	6 (13)	5 (11)	2 (5)	9 (28)	4 (13)	11 (9)	62 (14)
Unknown/missing	13 (19)	53 (87)	37 (79)	35 (76)	41 (93)	19 (59)	18 (56)	92 (77)	308 (68)
Stage at initial diagnosis, *n* (%)									
I	4 (6)	0	3 (6)	4 (9)	2 (5)	1 (3)	1 (3)	1 (<1)	16 (4)
II	0	4 (7)	5 (11)	3 (7)	4 (9)	2 (6)	0	6 (5)	24 (5)
III	4 (6)	13 (21)	3 (6)	3 (7)	7 (16)	3 (9)	1 (3)	4 (3)	38 (8)
I-III (NOS)	0	0	0	0	0	0	0	1 (<1)	1 (<1)
IV	48 (69)	32 (53)	9 (19)	16 (35)	10 (23)	9 (28)	23 (72)	57 (48)	204 (45)
Unknown/missing	14 (20)	12 (20)	27 (57)	20 (44)	21 (48)	17 (53)	7 (22)	50 (42)	168 (37)
ECOG PS at advanced/metastatic diagnosis, *n* (%)									
0	5 (7)	5 (8)	2 (4)	2 (4)	3 (7)	3 (9)	1 (3)	9 (8)	30 (7)
1	18 (26)	13 (21)	7 (15)	6 (13)	1 (2)	6 (19)	10 (31)	20 (17)	81 (18)
2	8 (11)	2 (3)	3 (6)	3 (7)	1 (2)	5 (16)	3 (9)	8 (7)	33 (7)
3	0	0	0	0	0	0	1 (3)	1 (<1)	2 (<1)
Missing	39 (56)	41 (67)	35 (75)	35 (76)	39 (89)	18 (56)	17 (53)	81 (68)	305 (68)
Metastasis status at advanced/metastatic diagnosis, *n* (%)									
No	45 (64)	24 (39)	10 (21)	19 (41)	26 (59)	19 (59)	19 (59)	77 (65)	239 (53)
Yes	25 (36)	37 (61)	37 (79)	27 (59)	18 (41)	13 (41)	13 (41)	42 (35)	212 (47)
*NTRK* gene fusion test type, *n* (%)									
NGS	58 (83)	55 (90)	35 (75)	33 (72)	40 (91)	23 (72)	23 (72)	96 (81)	363 (81)
FISH	5 (7)	1 (2)	0	0	2 (5)	0	3 (9)	5 (4)	16 (4)
IHC followed by confirmatory NGS	1 (1)	1 (2)	0	2 (4)	0	1 (3)	1 (3)	3 (3)	9 (2)
Missing/unknown	6 (9)	4 (7)	12 (26)	11 (24)	2 (5)	8 (25)	5 (16)	15 (13)	63 (14)
Days from advanced/metastatic diagnosis to first *NTRK* gene fusion test date									
Median (IQR)	10 (0-162)	14 (−4 to 281)	687 (9-1366)	13 (0-326)	739 (2-1875)	36 (0-129)	10 (0-85)	16 (0-234)	25 (0-459)
*NTRK* gene fusion, *n* (%)									
*NTRK1*	43 (61)	45 (74)	18 (38)	23 (50)	17 (39)	5 (16)	12 (38)	63 (53)	226 (50)
*NTRK3*	13 (19)	10 (16)	24 (51)	16 (35)	20 (46)	26 (81)	11 (34)	32 (27)	152 (34)
*NTRK2*	12 (17)	5 (8)	2 (4)	3 (7)	5 (11)	1 (3)	7 (22)	20 (17)	55 (12)
*NTRK1, NTRK2*	1 (1)	1 (2)	0	0	1 (2)	0	0	0	3 (<1)
Missing	1 (1)	0	3 (6)	4 (9)	1 (2)	0	2 (6)	4 (3)	15 (3)
Received radiation before advanced/metastatic disease, *n* (%)									
No	34 (49)	16 (26)	16 (34)	17 (37)	7 (16)	15 (47)	14 (44)	37 (31)	156 (35)
Yes	0	0	1 (2)	0	5 (11)	4 (13)	0	5 (4)	15 (3)
Missing	36 (51)	45 (74)	30 (64)	29 (63)	32 (73)	13 (41)	18 (56)	77 (65)	280 (62)
Received surgery before advanced/metastatic disease, *n* (%)									
No	33 (47)	13 (21)	8 (17)	10 (22)	5 (11)	10 (31)	13 (41)	38 (32)	130 (29)
Yes	1 (1)	3 (5)	9 (19)	7 (15)	7 (16)	9 (28)	1 (3)	6 (5)	43 (10)
Missing	36 (51)	45 (74)	30 (64)	29 (63)	32 (73)	13 (41)	18 (56)	75 (63)	278 (62)

CRC, colorectal cancer; ECOG PS, Eastern Cooperative Oncology Group performance status; GIST, gastrointestinal stromal tumor; IHC, immunohistochemistry; IQR, interquartile range; NGS, next-generation sequencing; NOS, not otherwise specified; NSCLC, non-small-cell lung cancer; *NTRK*, neurotrophic tyrosine receptor kinase.

a Other tumor types included appendix, bladder, brain, cholangiocarcinoma, esophageal, gallbladder, gastric, head and neck, kidney, liver, lung, melanoma, neuroendocrine, prostate, uterine, and cancer of unknown primary, together with GIST and unspecified sarcoma.

The *NTRK* gene fusions identified in this cohort involved *NTRK1* (*n =* 226, 50%), *NTRK3* (*n =* 152, 34%), *NTRK2* (*n =* 55, 12%), and *NTRK1* and *NTRK2* (*n =* 3, <1%); information was missing for 15 patients (3%). There were 106 *NTRK* gene fusion partners identified (Supplementary Table S4, available at https://doi.org/10.1016/j.esmorw.2026.100814). *NTRK1* had 64 unique fusion partners, *NTRK2* had 27, and *NTRK3* had 36. Nine fusion partners fused with all three *NTRK* genes, most commonly *ETV6* (19.5% of tumors), *TPM3* (10.3%), *INTRAGENIC* (10.1%), and *LMNA* (7.8%).

### Diagnostic and treatment patterns

*NTRK* gene fusions were identified predominantly by NGS (*n* = 363, 81%), less commonly by FISH (*n =* 16, 4%), or by immunohistochemistry followed by confirmatory NGS (*n =* 9, 2%); in 14% of patients (*n =* 63), the method was unknown (Table 1). In *NTRK* gene fusion-positive cancers, the median time from diagnosis of advanced/metastatic disease to *NTRK* gene fusion testing was 25 days (IQR 0-459).

The type of therapy patients received by line is shown in Figure 2, with the most common first LOT being chemotherapy (51%) and targeted small-molecule therapies (19%; i.e. afatinib, alectinib, entrectinib, erlotinib, imatinib, larotrectinib, lenvatinib, palbociclib, and sorafenib). Among patients who received a second LOT, targeted small-molecule therapy was the most common treatment (24%), followed by chemotherapy (16%); most patients did not reach a third or later LOT (Figure 2). Sankey plots of therapy for the first five LOTs by tumor type are shown in Supplementary Figures S3-S10 (available at https://doi.org/10.1016/j.esmorw.2026.100814). The plots demonstrate marked variability in treatment patterns by tumor type across the first five LOTs, reflecting treatment options available for each tumor type. Chemotherapy or targeted therapies frequently dominated initial treatment lines, while the proportion of patients who did not reach subsequent lines increased sharply after second-line treatment.

**Figure 2:**
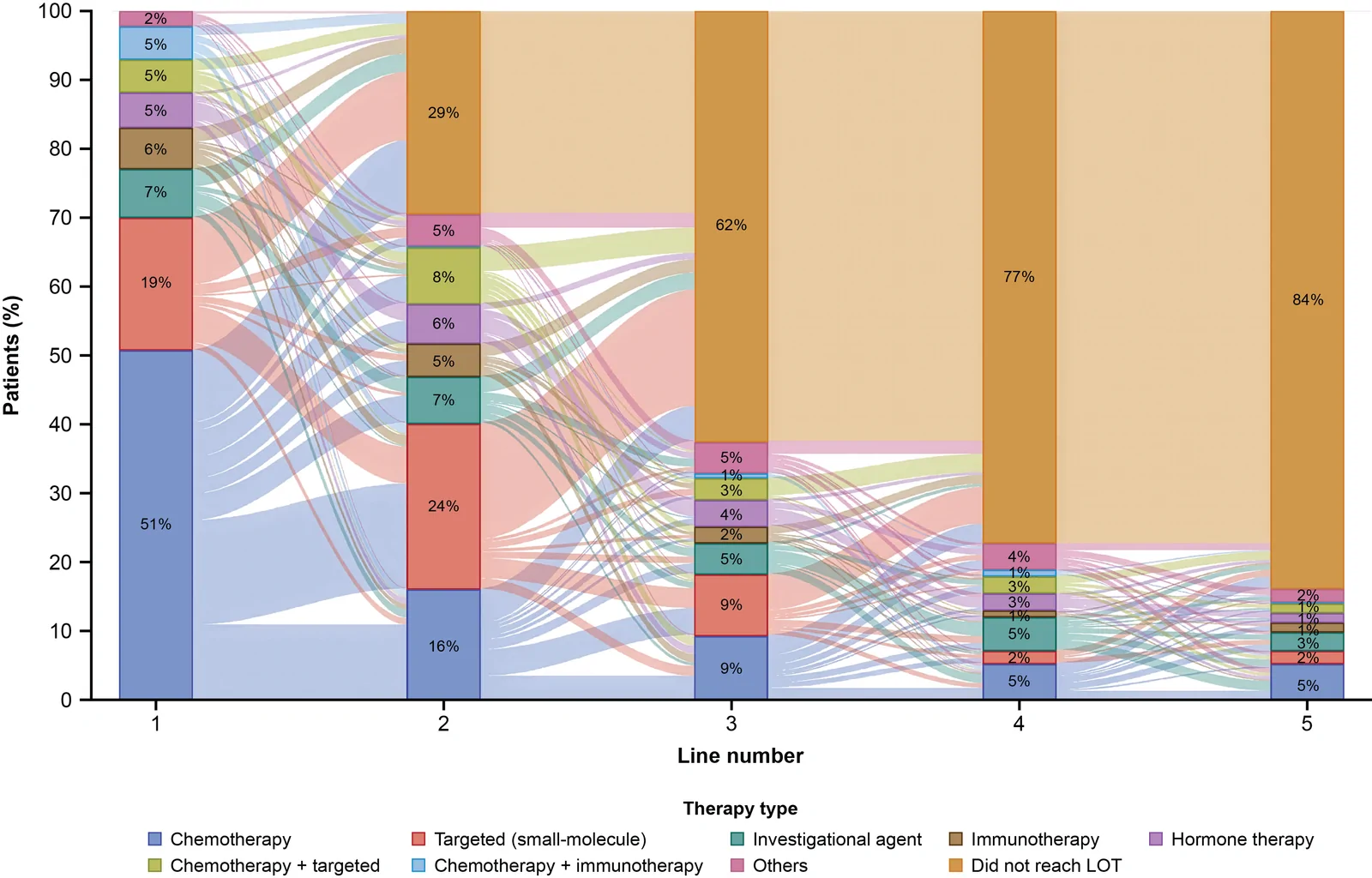
**Therapy type in patients with advanced/metastatic cancers by LOT number.** Targeted (small-molecule) therapies included afatinib, alectinib, entrectinib, erlotinib, imatinib, larotrectinib, lenvatinib, palbociclib, and sorafenib. LOT, line of therapy.

Overall, 172 (38.1%) patients received a TRK inhibitor. From 2018 onward, marking the US approval and availability of larotrectinib (the first TRK inhibitor approved, followed shortly by entrectinib), 135 of 276 (48.9%) patients who were diagnosed with an *NTRK* gene fusion received a TRK inhibitor. Overall, the median time from *NTRK* gene fusion testing to first TRK inhibitor administration was 118 days (IQR 31-335). From 2018 onward, the median time from *NTRK* gene fusion test to the initiation of TRK inhibitor therapy was 105 days (IQR 30-240).

### Clinical outcomes

The left truncation median rwOS from start of any first-line systemic therapy for advanced/metastatic cancers ranged from 41 months (95% CI 25-50) for breast cancer to 11 months (95% CI 8-17) for pancreatic cancer (Figure 3A). The median DOT for the first LOT for advanced/metastatic cancers varied between 3 months (95% CI 2-4, IQR 1-6) for soft-tissue sarcoma and 10 months (95% CI 3-13, IQR 2-20) for thyroid cancer (Figure 3B). The median DOT of subsequent LOTs was similar to the second and third lines, and reduced with additional LOTs (Supplementary Figure S2, available at https://doi.org/10.1016/j.esmorw.2026.100814). The median TTNT from first LOT was longest for patients with thyroid cancer [19 months (95% CI 13-93, IQR 12-93)] and shortest for breast cancer [6 months (95% CI 4-10, IQR 4-12)] and pancreatic cancer [6 months (95% CI 6-12)] (Figure 3C). The median rwPFS for first LOT was similarly greatest for thyroid cancer, being 15 months (95% CI 10-31, IQR 7-42), and was least for salivary gland cancer and pancreatic cancer, being 6 months (95% CI 4-11, IQR 3-37) and 7 months (95% CI 5-11, IQR 3-11), respectively (Figure 3D). The rwRR by tumor type is shown in Supplementary Table S5 (available at https://doi.org/10.1016/j.esmorw.2026.100814).

**Figure 3:**
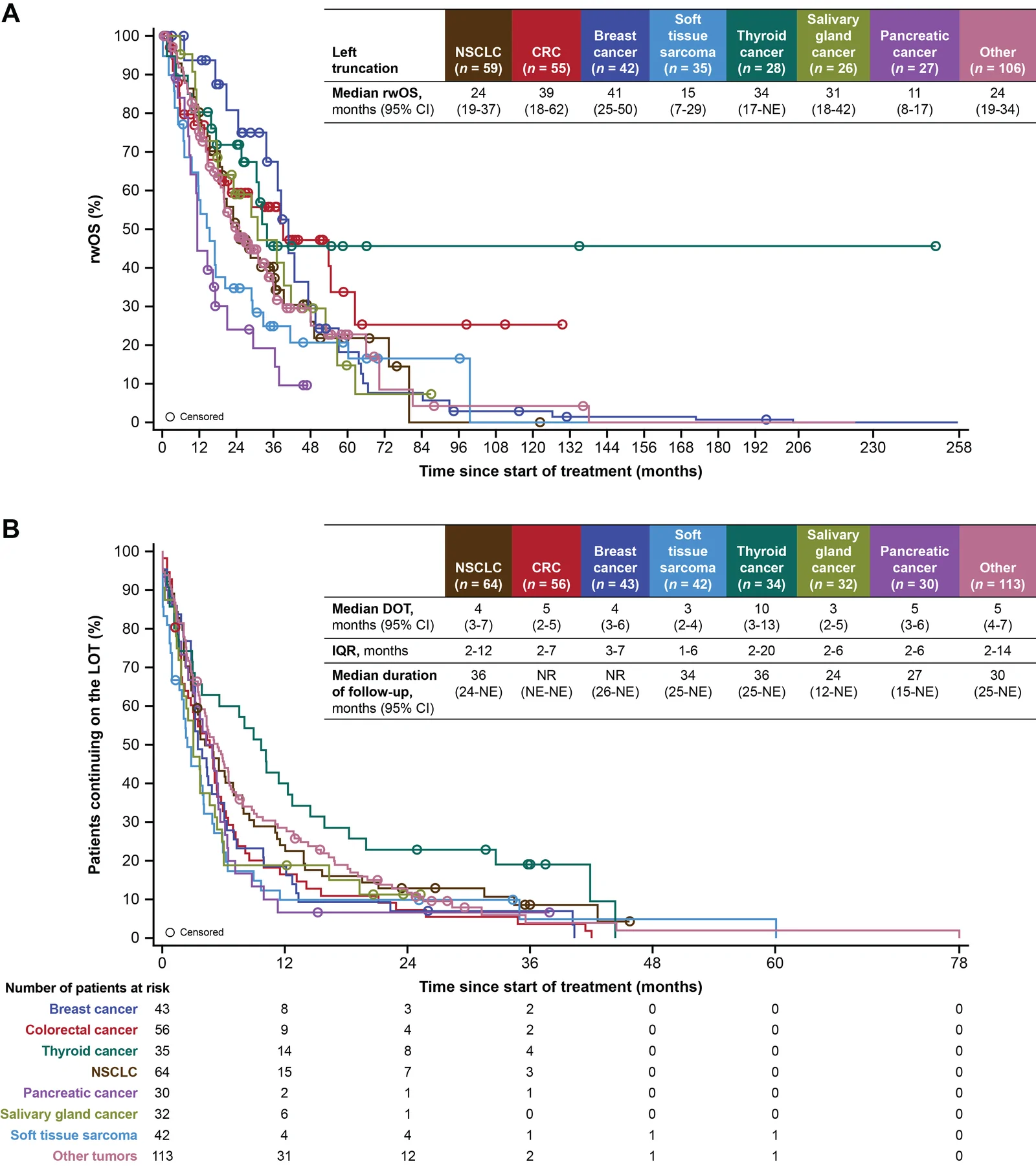

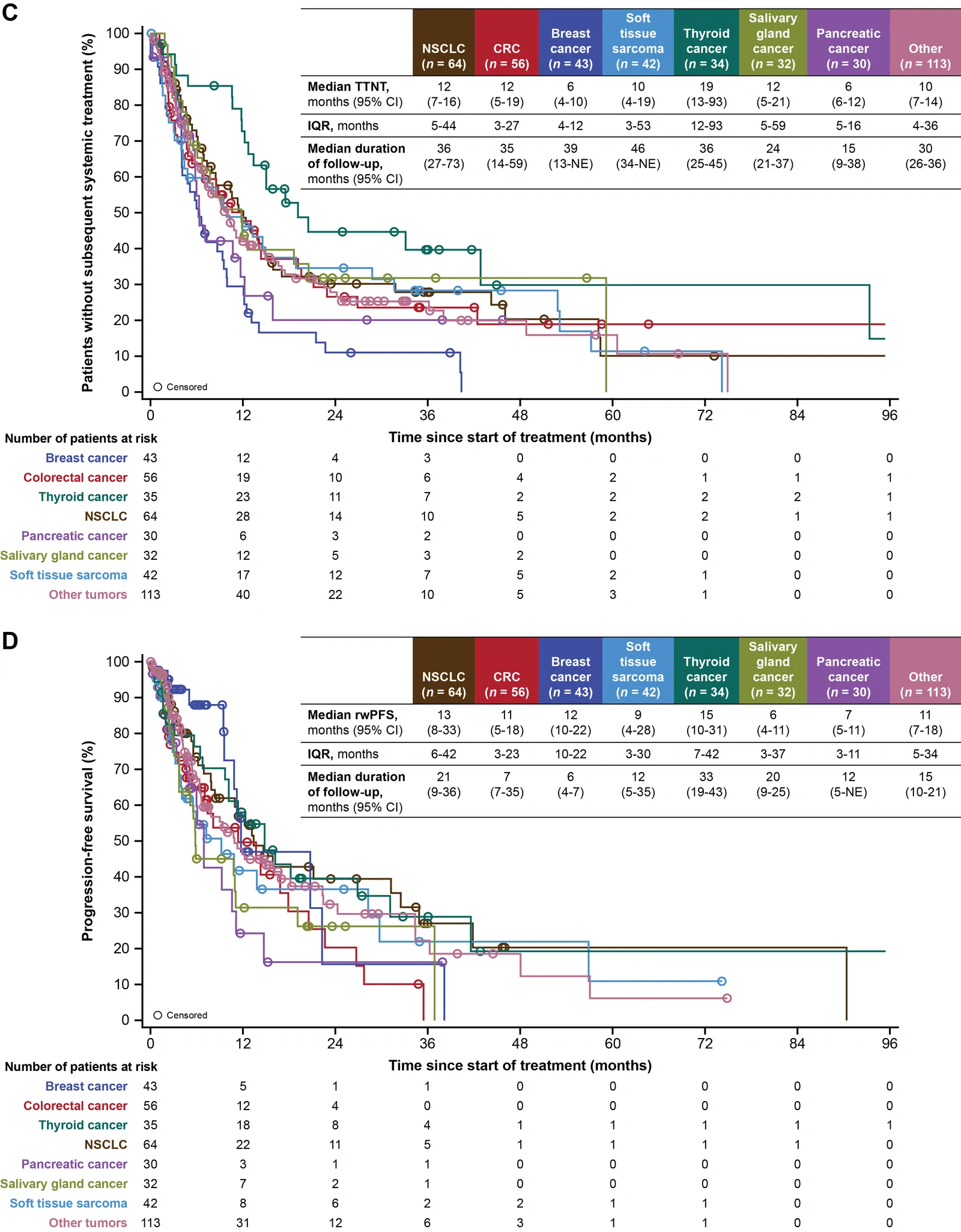
**Kaplan–Meier curves of rwOS (A), DOT (B), TTNT (C), and rwPFS (D) for first LOT by tumor type in patients with advanced/metastatic cancers.** Left truncation analysis was applied in estimating rwOS for adult patients only. CI, confidence interval; CRC, colorectal cancer; DOT, duration of therapy; IQR, interquartile range; LOT, line of therapy; NE, not estimable; NSCLC, non-small-cell lung cancer; rwOS, real-world overall survival; rwPFS, real-world progression-free survival; TTNT, time to next treatment.

The median rwOS (left truncation), DOT, TTNT, and rwPFS by LOT are shown in Supplementary Figure S2 (available at https://doi.org/10.1016/j.esmorw.2026.100814). Survival outcomes decreased with subsequent treatment lines, although the second LOT, where there was greater use of targeted therapies (Figure 2), showed a prolonged median DOT and TTNT as compared with other LOTs.

## Discussion

In this study, 451 patients with advanced/metastatic TRK fusion solid tumors were identified, representing the largest real-world cohort of this patient population to date. Additionally, by incorporating datasets from both academic (AACR-GENIE and ORIEN) and community (Cardinal Health, FH-FMI CGDB, Global Chart Review Study) settings, this study reflects diverse clinical practices, enhancing the generalizability of the results.

An expected finding from this study was that NGS testing was the most used method for detection of *NTRK* gene fusions (81% of patients). A recent US-based, retrospective, observational study (*N =* 55) conducted at six academic cancer centers in a similar population reported that 98% of their study sample used NGS as the primary method to detect *NTRK* gene fusions.^22^ A one-time physician questionnaire, combined with a retrospective, multisite patient chart review across 28 US oncology practices between 1 January 2016 and 31 December 2019, observed that 96% of the 148 patients had *NTRK* gene fusion status determined using NGS.^24^ The somewhat lower rate of NGS leading to diagnosis of an *NTRK* gene fusion in our dataset probably reflects the more heterogeneous nature of this globally pooled cohort; access to comprehensive molecular profiling and molecular diagnostic testing evolved during the study period, and testing practices may differ across health care systems because of variations in availability, implementation, and reimbursement. Consequently, the testing patterns observed in this study should be interpreted within the context of the study period and participating data sources. The use of NGS over less comprehensive alterations is key, as concurrent alterations or signatures can be identified. In some contexts (e.g. rare primary resistance mediated by non-TRK activation, or CRC harboring a microsatellite instability-high phenotype), characterizing this landscape is crucial.^25^

The current study reported a median time from advanced/metastatic diagnosis to the first *NTRK* gene fusion test of 25 days (IQR 0-459), although variability was high. The most probable cause of this variability is the fundamental difference between the clinical and diagnostic management of the various tumor types in this cohort. While some tumor types [i.e. NSCLC, pancreatic cancer, soft tissue sarcoma, CRC, and salivary gland cancer (median time from diagnosis to test result ranging from 10 to 36 days)] were tested relatively early, others [i.e. thyroid and breast cancers (median time from diagnosis to test result of 687 and 739 days, respectively)] were tested much later. These different patterns reflect the heterogeneity of comprehensive molecular profiling approaches, conditioned by the availability of other signatures (e.g. druggable oncogenes and tumor mutational burden) that match patients to approved therapies. In this setting, it has to be acknowledged that the European Society for Medical Oncology and American Society of Clinical Oncology now recommend comprehensive genomic profiling across a broad spectrum of advanced cancers at diagnosis.^26^^,^^27^

Also in the current study, only half (48.9%) of the patients who tested positive for an *NTRK* gene fusion from 2018 onward received a TRK inhibitor. While an adoption rate approaching 50% appears relatively encouraging, especially for tumor types such as NSCLC and CRC where multiple established first-line treatment options exist, this also suggests an educational need regarding the utility of targeted therapy, or the need to test for these fusions earlier while patients remain fit. The median time from *NTRK* gene fusion testing to first TRK inhibitor administration was 118 days (IQR 31-335). Our study was not designed to assess clinical decision-making ahead of the initiation of TRK inhibitor treatment. As for differences in the testing patterns, these may be explained by the heterogeneity of the cohort, the resulting differences in clinical management, and the varying clinical needs for initiation of treatment with TRK inhibitor therapy. Future research may help provide insight into why some patients identified with *NTRK* gene fusions do not receive TRK inhibitor therapy.

The left truncation median rwOS varied widely across tumor types, from 11 months (95% CI 8-17) for pancreatic cancer to 41 months (95% CI 25-50) for breast cancer. Because the long median time from advanced/metastatic diagnosis to *NTRK* gene fusion testing for breast and thyroid cancers could introduce survival or immortal time bias, drastically overestimating true real-world survival time, left truncation was applied to the rwOS analysis to account for delayed entry into the risk set at the time of genomic sequencing. Median rwOS also decreased as the number of prior LOTs increased, from 28 months (95% CI 21-32) for first LOT to 8 months (95% CI 6-12) for fifth LOT. A previous study by Hibar et al.^28^, in 28 patients with locally advanced/metastatic TRK fusion-positive solid tumors from FHI–FMI CGDB (1 January 2011 to 31 December 2019) who had received no TRK inhibitor during their patient journey, reported a median OS from advanced/metastatic diagnosis of 10.2 months (95% CI 7.2-14.1). However, due to the very small sample size and utilization of real-world data before the advent of TRK inhibitors in this study,^28^ it is challenging to compare these rwOS data with those from our current study.

For additional historical context from the clinical-trial setting, a pooled retrospective growth modulation index (GMI) analysis of larotrectinib (three phase I/II trials; data cut-off July 2021) reported a median progression-free survival on larotrectinib of 19.6 months versus a median time to progression on prior therapy of 3.0 months.^29^ In this study, 47 (69%), 29 (59%), and 47 (80%) patients with 1, 2, or ≥3 prior lines, respectively, had a GMI of ≥1.33 while receiving larotrectinib. The reported GMI of ≥1.33 indicated clinically meaningful intrapatient benefit in larotrectinib trial populations.^29^

Thus, there is a need for further studies on the effectiveness of TRK inhibitors in real-world settings as treatment options evolve. In a retrospective study of patients with solid tumors sequenced through the Veterans Affairs National Precision Oncology Program (VA NPOP), only 0.12% with solid tumors were identified as having *NTRK* gene fusions or rearrangements,^17^ which is lower than rates found in prior real-world studies.^2^^,^^13^ Among 12 veterans with *NTRK* gene fusions or rearrangements who were treated with TRK inhibitors in the VA NPOP study, no objective responses were observed,^17^ illustrating different findings compared with those from clinical trials. However, the mix of patients with *NTRK* gene fusions and those with *NTRK* gene rearrangements in the VA NPOP study demonstrates the importance of improved molecular profiling and interpretation of results in real-world clinical settings to correctly identify patients with *NTRK* gene fusions most likely to benefit from TRK inhibitor therapy.^30^ Moreover, only four of 12 patients who received a TRK inhibitor were identified with canonical *NTRK* gene fusions and these were the only patients reported to have stable disease with TRK inhibition, supporting the hypothesis that the noncanonical fusions present in the remaining two-thirds of patients were not activating events.^30^ The VA NPOP study was further limited by the small sample size, the use of tumor DNA sequencing rather than RNA sequencing, and the definition of response.^17^^,^^30^

The current study was descriptive in nature; therefore, causal inferences cannot be drawn from these findings. While TRK inhibitors were introduced and testing options became more widely available later in the study period, earlier data were included to understand treatment patterns and trends over time in the study population. As such, the changing clinical landscape over the duration of the study period may be reflected in the real-world treatment patterns and outcomes that were observed. Additionally, the use of multiple data sources may have led to some level of missing data, as well as variations in reliability of data, data collection methods, and patient follow-up duration. Notably, genomic data were only available from the AACR-GENIE and FH-FMI CGDB data sources. Also, the study population was predominantly White (76%), which may limit generalizability to more racially diverse populations. Furthermore, as the sample size for pediatric patients was small (all patients with initial cancer diagnosis, *N =* 32; patients with advanced/metastatic disease, *N =* 15), the analyzed patient population may not be representative of the general pediatric population with TRK fusion solid tumors. Also, there was great diversity in LOTs. Finally, the study did not report individual adverse events or safety data as the data sources used were not designed to record such information. Missing data generally occurred randomly across databases, reflecting a recognized limitation of real-world data.^31^ Furthermore, missingness often reflected the clinical relevance of specific variables to individual cancer subtypes. For example, smoking status is highly relevant to NSCLC, whereas breast, thyroid, and salivary gland cancers are not strongly associated with smoking.^32^, ^33^, ^34^

This study is the largest integrated real-world cohort describing clinical characteristics, testing and treatment patterns, and clinical outcomes in patients with TRK fusion solid tumors to date. The findings provide a more comprehensive picture of TRK fusion solid tumors in the age of TRK-targeted therapy.
