# Supplementary material for: Real-world characteristics, testing patterns, and outcomes in patients with tropomyosin receptor kinase fusion cancer from a large pooled clinicogenomic database
Source: ESMO Real World Data Digit Oncol. 2026 Nov 5;14:100814. doi: 10.1016/j.esmorw.2026.100814 (PMC13753593; doi:10.1016/j.esmorw.2026.100814)
Supplement: Supplementary file 1 [file mmc1.docx]

**SUPPLEMENTARY MATERIALS**

**SUPPLEMENTARY METHODS**

***Data sources***

Given the rarity of patients with TRK fusion cancer, identification of relevant real-world data sources can be challenging.^1^ Thus, data sources were selected for use in this study based on the availability of patient data, genomic testing and accessibility of patient-level data.

The American Association of Cancer Research Genomics Evidence Neoplasia Information Exchange database is an international cancer registry of real-world data-sharing between 19 of the leading cancer centers in the world. The registry aggregates and links clinical-grade, next-generation cancer genomic sequencing data with clinical outcomes obtained during routine medical practice from patients with cancer treated at these institutions.^2^

The Cardinal Health study was a Bayer-sponsored retrospective cohort study of patients with tumors with TRK fusions in the United States (US).^3^

The US-based Flatiron Health–Foundation Medicine Clinico-Genomic Database (FH-FMI CGDB) contains de-identified data originating from approximately 280 US cancer clinics.^4^ Retrospective, longitudinal clinical data were derived from electronic health record data, comprising patient-level structured and unstructured data curated via technology-enabled abstraction, and were linked to genomic data derived from FMI comprehensive genomic profiling (CGP) tests in the FH-FMI CGDB by de-identified, deterministic matching.^5^ Genomic alterations were identified via CGP of >300 cancer-related genes using FMI’s next-generation sequencing (NGS) test(s).^6-8^The US-based Flatiron Health–Foundation Medicine Clinico-Genomic Database (FH-FMI CGDB) contains de-identified data originating from approximately 280 US cancer clinics.^4^ Retrospective, longitudinal clinical data were derived from electronic health record data, comprising patient-level structured and unstructured data curated via technology-enabled abstraction, and were linked to genomic data derived from FMI comprehensive genomic profiling (CGP) tests in the FH-FMI CGDB by de-identified, deterministic matching.^5^ Genomic alterations were identified via CGP of >300 cancer-related genes using FMI’s next-generation sequencing (NGS) test(s).^6-8^ The study included 23 matched patients from a multi-tumor cohort, which included 5 tumor types (non-small cell lung cancer [NSCLC], colorectal cancer [CRC], thyroid cancer, soft tissue sarcoma, and salivary gland carcinoma) with confirmed *NTRK* gene fusions. The Oncology Research Information Exchange Network data sources include electronic medical records, pharmacy records, and cancer registries from 6 academic centers led by the University of Utah.^9^

The global chart review included 6 sites across 6 countries (Australia, Canada, Italy, Taiwan, the United Kingdom, and the US). A clinical site survey was designed and conducted to identify adult patients (≥18 years) with solid tumors (NSCLC, CRC, thyroid cancer, soft tissue sarcoma, and salivary gland carcinoma) harboring neurotrophic tyrosine receptor kinase (*NTRK*)*1*, *NTRK2*, or *NTRK3* gene fusions at 915 clinical sites.^10^ The initial survey questionnaire (administered from September 8, 2021 to October 10, 2021) was designed through an iterative internal review process and was sent to clinical sites up to 3 times to elicit a response (original survey email/outreach along with 2 follow-up emails/outreaches if no response was received after initial outreach; each follow-up email was sent ~1 week after the previous unanswered survey). Following the initial survey outreach, feedback from clinical sites was utilized to update the survey, which was simplified and redesigned again through an iterative internal review process to increase responsiveness from clinical sites. The focus of the simplified survey was also broadened to include both adult and pediatric patient populations. The simplified survey (administered from February 1, 2022 to March 28, 2022) was sent to clinical sites that had not responded to the first survey up to 3 times to elicit a response (simplified survey email/outreach along with 2 follow-up emails/outreaches). The simplified survey was composed of 8 multiple-choice and free-text questions that assessed the type of patients with cancer treated at each institution (e.g. adult, pediatric, or both), whether the institution was conducting *NTRK1–3* gene fusion molecular diagnostic testing, and whether the institution had an estimate of the number of patients identified with *NTRK1–3* gene fusions over the last 12 months and 5 years. The survey then aimed to quantify the number of patients with *NTRK1–3* gene fusions by tumor type. As Bayer was the sponsor of this study, the clinical sites included in the survey were compiled from 3 main sources: sites considered for inclusion in the larotrectinib clinical trial program, clinical sites where patient(s) were treated with larotrectinib in the real-world setting, and sites identified from a systematic literature review on epidemiology of *NTRK* gene fusions.^11^ The clinical site list was then sent to Bayer medical science liaisons globally for help in finalizing the list with clinical site contact information. Sites were contacted via email, with no more than 6 contact attempts made per site or coordinator. To qualify for the survey, respondents had to opt in to the survey after reading the introduction and study purpose, read and agree to the Protection of Personal Data and Data Privacy Rights statements (ex-US only), and conduct genomics testing on tissue samples for *NTRK1–3* gene fusions in their practice. Reasons for site disqualification included non-response, no patients reported to be *NTRK* gene fusion-positive, lack of testing for *NTRK* gene fusions, inability to discern appropriate contact, and opting out of the survey. Survey responses were evaluated on a weekly basis, and strategies to boost response rates, in addition to the simplified clinical sites survey, were implemented, including 2 email reminders sent to non-responders and faxable options for sites requesting a different survey method. The final clinical sites survey outreach responses and non-responses were collated to determine the frequency of responders in the US and ex-US.

***Deduplication of patient-level data across real-world sources***

In the first stage, patient records were exactly matched (1:1) based on the following variables: year of birth, sex, tumor type, and date of cancer diagnosis. The second stage involved a manual review of the following variables: region/site, date of treatment initiation, and treatment sequencing pattern. If the same patient existed in multiple data sources, and the follow-up started from the same line of therapy across the sources, then the observation with the longest follow-up period was used in the pooled analyses, and the duplicate observation(s) in the other source(s) was removed, resulting in a deduplicated dataset. Further information regarding data collection methods has been previously published.^1^

***Exploratory analysis of all patients with TRK fusion cancers***

For the wider exploratory cohort of all patients with a diagnosis of TRK fusion cancer during the study period, regardless of disease stage, the index date was the date of initial cancer diagnosis (Figure 1). The baseline period was defined as the period that began on or up to 3 months (90 days) prior to the initial index date. The follow-up period was defined from the initial index date to the date of last observation, the end of the study period, or death, whichever occurred first. Patient demographics and clinical characteristics were collected. Co-occurring genomic alterations were recorded using descriptive statistics reported as frequencies and percentages.

**SUPPLEMENTARY RESULTS**

***Patient characteristics in patients with an initial diagnosis of cancer***

A total of 512 patients (480 adult and 32 pediatric patients) received an initial diagnosis of cancer (regardless of disease stage) during the study period. Baseline demographics and clinical characteristics by tumor type are shown in Supplementary Table 6. Tumor types included NSCLC (*n =* 72), CRC (*n =* 68), thyroid cancer (*n =* 63), soft tissue sarcoma (*n =* 56), breast cancer (*n =* 47), salivary gland cancer (*n =* 36), pancreatic cancer (*n =* 32), and other cancers (*n =* 138). The median age was 58 years (range 0–92). There were 274 female patients (54%), 237 male patients (46%), and information was missing for 1 patient (<1%).

The test types used to identify *NTRK* gene fusions were NGS (*n =* 414; 81%), fluorescence in situ hybridization (FISH; *n =* 16, 3%), immunohistochemistry followed by confirmatory NGS (*n =* 9, 2%), and an unknown method/method information missing (*n =* 73; 14%). The *NTRK* gene fusions involved *NTRK1* (*n =* 255; 50%), *NTRK3* (*n =* 174; 34%), *NTRK2* (*n =* 56; 11%), *NTRK1* and *NTRK2* (*n =* 3; <1%), and information was missing for 24 patients (5%). The summary of baseline demographics and clinical characteristics in adult and pediatric patients is shown in Supplementary Table 7. There were 118 *NTRK* gene fusion partners identified (Supplementary Table 8).

***Exploratory analysis of all patients with TRK fusion cancer***

Genomic data were only available from the AACR GENIE (*N =* 255) and FH-FMI CGDB (*N =* 98) data sources. Co-occurring genomic alterations present in ≥10% patients are shown in Supplementary Figure 11. The most common high and medium impact genomic alterations (i.e. mutations with known or likely functional change, though they may not necessarily be oncogenic or druggable) co-occurring in patients with *NTRK* gene fusions overall were *TP53* (*n =* 113; 32%), *CDKN2A* (*n =* 52; 15%), *CDKN2B* (*n =* 48; 14%), *RNF43* (*n =* 28; 8%), *TERT* (*n =* 25; 7%), *KMT2D* (*n =* 23; 7%), *PIK3CA* (*n =* 23; 7%), *KRAS* (*n =* 18; 5%), *APC* (*n =* 18; 5%), and *ARID1A* (*n =* 18; 5%).

**Supplementary Table 1. ESMO GROW checklist**

| **Recommendations** | **Fully reported** | **Partially reported** | **Not reported** | **Not applicable** |
| --- | --- | --- | --- | --- |
| **1. Title** |  |  |  |  |
| 1.1: Concisely include relevant key terms referring to the study type, study population, objectives, data sources and outcomes, depending on the study. Consider including the terms ‘real-world’ or ‘observational’ | X |  |  |  |
| **2. Introduction** |  |  |  |  |
| 2.1: Explain the scientific rationale for the research question(s), providing concise background information on previous core evidence from systematic reviews, meta-analyses, clinical trials and/or real-world evidence studies | X |  |  |  |
| 2.2: Identify the gaps in evidence and explain why and how they can be suitably addressed by real-world evidence research. Specify the new evidence that is expected from the current study | X |  |  |  |
| 2.3: Briefly introduce the aim(s) of the study | X |  |  |  |
| **3. Methods** |  |  |  |  |
| ***Study objective(s), design, data sources and variables*** |  |  |  |  |
| 3.1: Provide the study research question(s) including a description of the patients or the object under study and the target outcome(s) | X |  |  |  |
| 3.2: Provide the study objective(s) and consider classifying the type of research as descriptive and/or analytical (explanatory or predictive) | X |  |  |  |
| 3.3: Provide relevant information to describe and classify the study design used to address the research question | X |  |  |  |
| 3.4: Give a clear definition of the eligibility criteria used to select the patients or objects under study, particularly regarding cancer-related aspects | X |  |  |  |
| 3.5: Report the specific type and purpose of real-world data source(s) used, providing a detailed description and the reason(s) why the source was considered appropriate for the study objectives | X |  |  |  |
| 3.6: When multiple real-world data sources are used, provide details on interoperability, including identification of duplicated cases or data linkage from separate databases | X |  |  |  |
| 3.7: Provide details and timings of source and study data management. Consider specifying methods of raw data collection, updates and completeness, data extraction, cleaning and/or quality controls and validation | X |  |  |  |
| 3.8: Provide core details on database and/or study registration, governance, ownership, metadata and full data accessibility in the main text or supplementary material | X |  |  |  |
| 3.9: Identify the data source of each core variable, its definition, if the variable was derived or coded, and describe how the derivation or coding was conducted and validated | X |  |  |  |
| 3.10: Specify the time points of core variables in relation to the cancer disease trajectory | X |  |  |  |
| 3.11: Provide a complete list of core variables included in the study. Variables can be grouped as baseline characteristics, exposure and outcomes or endpoints | X |  |  |  |
| 3.12: For biomarker-related studies, provide details on biomarker description, timing, and methods of assessment and analytical validation |  |  |  | X |
| ***Statistical analysis and artificial intelligence methods*** |  |  |  |  |
| 3.13: Summarise the main aspects of the statistical analysis | X |  |  |  |
| 3.14: When applicable, provide details on the pre-planned sample size requirements and power of the study |  |  |  | X |
| 3.15: Specify the pre-planned strategies to identify and mitigate the main sources of bias | X |  |  |  |
| 3.16: Clearly distinguish prespecified from post hoc analyses, especially for subgroup analyses | X |  |  |  |
| 3.17: Provide information on internal and external validity, as well as any sensitivity analyses | X |  |  |  |
| 3.18: For analytical studies, the full version of the statistical analysis plan should be provided in the supplementary material, including a brief explanation of any amendments |  |  |  | X |
| 3.19: When applicable, specify which machine learning, deep learning or alternative artificial intelligence method has been used |  |  |  | X |
| 3.20: When reporting real-world data analysis with artificial intelligence (e.g. machine learning and deep learning) algorithms, include comprehensive aspects on data pre-processing techniques, feature engineering strategies and model development |  |  |  | X |
| 3.21: Address the artificial intelligence model explainability and interpretability, and present the plan for integration into clinical practice, if applicable |  |  |  | X |
| 3.22: When applicable, briefly describe the multidisciplinary team required for the study and explain how these needs were met |  |  |  | X |
| **4. Results** |  |  |  |  |
| 4.1: Provide number of cases excluded or nonparticipating and reasons at each stage of sample selection, as well as numbers lost to follow-up. Compare the cases excluded with those included in the analyses. Illustrate this with a flowchart | X |  |  |  |
| 4.2: Describe the baseline characteristics of the cases included (e.g. clinico-demographic and tumour characteristics). The baseline characteristics of different groups under analysis should be compared, if applicable | X |  |  |  |
| 4.3: Report the results of the primary analysis of study outcomes. Briefly describe the results of exploratory analyses if relevant (prespecified and/or post hoc). Provide details of how readers can access the full results | X |  |  |  |
| **5. Discussion and conclusions** |  |  |  |  |
| ***Discussion*** |  |  |  |  |
| 5.1: Summarise the core results that address the primary research question(s) and objectively discuss the data in relation to the best available evidence on the topic. Avoid a convenient selection of literature to support a point | X |  |  |  |
| 5.2: Discuss the strengths and limitations of the current study, including the main biases, how the strategies applied contributed to bias avoidance or mitigation, and, if applicable, in which direction the authors estimate that residual bias may influence the core results of the study | X |  |  |  |
| 5.3: Discuss the generalisability of the study results and their potential implications for clinical practice, health policies or public health and for the generation of hypotheses for future research | X |  |  |  |
| ***Conclusions*** |  |  |  |  |
| 5.4: Provide a balanced summary of core results relating to the primary research question and the main implications for clinical practice, health policies and/or public health. Suggest further research considering the remaining unmet needs and limitations from the reported study | X |  |  |  |
| **6. Final considerations** |  |  |  |  |
| 6.1: Specify all relevant study sponsorship(s) as well as direct and/or indirect or in-kind funding | X |  |  |  |
| 6.2: Specify all relevant acknowledgements, author disclosures, individual contributions and other final considerations as per journal regulations | X |  |  |  |

**Supplementary Table 2. Outcomes definition**

| **Variable** | **Period of assessment** |
| --- | --- |
| **Demographic characteristics**   - Age at initial diagnosis - Sex - Race - Ethnicity - Country/region of residence - Smoking status | Baseline period (at main index date^†^) |
| **Clinical characteristics**   - Advanced or metastatic diagnosis   - Tumor type   - Date of diagnosis   - Stage   - ECOG PS   - Metastasis - Diagnosis of *NTRK* gene fusion   - Date of diagnosis, defined as date of the test performance used to determine patient’s *NTRK* gene fusion status   - Type: *NTRK1*/*2*/*3* or unknown   - Test type: NGS, IHC, RT-PCR, FISH, other - *NTRK* gene fusion partner - Therapies received prior to advanced or metastatic disease, dates   - Surgery received prior to advanced or metastatic disease   - Radiation received prior to advanced or metastatic disease | Baseline period (at main index date^†^) |
| ***NTRK* gene fusion testing methods**   - NGS - FISH - RT-PCR | Follow-up period (from main index date^†^) |
| **Time to first *NTRK* gene fusion test performance**   - Time in days from main index date to first *NTRK* gene fusion test performance date (i.e. test order date or specimen collection date depending on available source data) | Follow-up period |
| **Time to initial TRK-targeted therapy treatment**   - Time in days from first *NTRK* gene fusion test result to initial TRK-targeted therapy treatment   - Initial TRK-targeted therapy was defined as the first *NTRK* inhibitor treatment following the main index date   - Patients who did not receive TRK-targeted treatment were censored at end of follow-up | Follow-up period from main index date |
| **LOT**   - Defined as distinct treatment combinations of systemic therapy, which includes chemotherapy, immunotherapy, and targeted medicine   - First line was defined as the first systemic therapy after diagnosis of advanced stage or metastatic diagnosis, and subsequent therapies were designated as second line, third line, etc. | Follow-up period from main index date |
| **DOT**   - Time from initiation of LOT until permanent treatment discontinuation, defined as the end of a LOT followed by another LOT, therapy discontinued with physician’s note, discontinued for a pre-defined gap period (e.g. >90 days), or death for any reason   - For patients who discontinued the therapy of interest, DOT was measured as time from LOT initiation date to date of discontinuation   - For patients who were still on the therapy of interest at the end of follow-up, patients were censored at the date of last known activity for the specific LOT, defined as the last administration or non-cancelled order of a drug contained within the treatment regimen | Follow-up period from main index date |
| **TTNT**   - Time from first LOT until date of initiation of next LOT or death   - For patients who received a subsequent LOT, TTNT was measured as time from first LOT to the date of initiation of the next LOT   - For patients who did not receive a subsequent treatment (continuing current treatment at the time of the analysis or loss of follow-up not due to confirmed death), patients were censored at the date of last known activity, defined as the last administration or non-cancelled order of a drug contained within the treatment regimen   - For patients who did not receive a subsequent treatment and had a confirmed death, TTNT was measured as time from first LOT to date of death | Follow-up period from main index date |
| **Type of therapy**   - Chemotherapy - Targeted therapy - Immune checkpoint inhibitor | Follow-up period from main index date |
| **Systemic therapy**   - Received systemic therapy - Did not receive systemic therapy | Follow-up period from main index date |
| **rwOS**   - Time from initiation of each treatment line to death   - Patients without a death event were censored at either last recorded clinical activity or end of follow-up period, whichever occurred latest | Follow-up period after main index date |
| **rwPFS**   - Time from initiation of each LOT to date of progression or date of death (death must occur within 30 days after last assessment or within 30 days after first LOT end date)   - For patients who had no subsequent LOT, if death occurred more than 30 days after last assessment date or first LOT end date (if no assessment information available) censoring occurred at 30 days after the last assessment or first LOT end date   - For patients who had subsequent LOT, and for whom no progression occurred before subsequent treatment (patients may have progressed or died after subsequent treatment), censoring occurred at last assessment date before subsequent LOT start date or 1 day prior to subsequent LOT start date   - For patients who had no subsequent LOT, and for whom no progression or death occurred, censoring occurred at the last assessment date or first LOT end date   - For patients who had no subsequent LOT, and for whom no progression or death occurred, and for whom the first LOT end date was missing, censoring occurred at the last assessment date or last known alive date | Follow-up period after main index date |
| **rwRR**   - Proportion of patients with an assessment of real-world complete response or partial response after initiation of line-specific treatment among all patients | Follow-up period after main index date |

^†^Also assessed at/from the alternative index date. DOT, duration of therapy; ECOG PS, Eastern Cooperative Oncology Group performance status; FISH, fluorescence in situ hybridization; IHC, immunohistochemistry; LOT, line of therapy; NGS, next-generation sequencing; *NTRK*, neurotrophic tyrosine receptor kinase; RT-PCR, reverse transcriptase polymerase chain reaction; rwOS, real-world overall survival; rwPFS, real-world progression-free survival; rwRR, real-world response rate; TTNT, time to next treatment; TRK, tropomyosin receptor kinase.

**Supplementary Table 3. Baseline and clinical characteristics in adult and pediatric patients with advanced/metastatic cancers**

|  | **Age <18 years**  ***n =* 15** | **Age ≥18 years**  ***n =* 436** | **Total**  ***N =* 451** |
| --- | --- | --- | --- |
| **Sex**, n (%) |  |  |  |
| Female | 9 (60) | 223 (51) | 232 (51) |
| Male | 5 (33) | 213 (49) | 218 (48) |
| Missing | 1 (7) | 0 | 1 (<1) |
| **Age at advanced/metastatic diagnosis,** years |  |  |  |
| Median (range) | 3 (0–17) | 60 (19–85) | 60 (0–85) |
| **Race**, n (%) |  |  |  |
| White | 5 (33) | 336 (77) | 341 (76) |
| Asian | 1 (7) | 31 (7) | 32 (7) |
| Black | 2 (13) | 28 (6) | 30 (7) |
| Other | 4 (27) | 24 (6) | 28 (6) |
| Unknown/missing | 3 (20) | 17 (4) | 20 (4) |
| **Ethnicity**, n (%) |  |  |  |
| Not Hispanic or Latino | 9 (60) | 299 (69) | 308 (68) |
| Hispanic or Latino | 4 (27) | 31 (7) | 35 (8) |
| Unknown/missing | 2 (13) | 106 (24) | 108 (24) |
| **Region**, n (%) |  |  |  |
| South | 0 | 41 (9) | 41 (9) |
| West | 0 | 34 (8) | 34 (8) |
| Northeast | 0 | 24 (6) | 24 (5) |
| Midwest | 0 | 11 (3) | 11 (2) |
| Unknown/missing | 15 (100) | 326 (75) | 341 (76) |
| **Smoking status**, n (%) |  |  |  |
| History of smoking | 0 | 81 (19) | 81 (18) |
| No history of smoking | 0 | 62 (14) | 62 (14) |
| Unknown/missing | 15 (100) | 293 (67) | 308 (68) |
| **Tumor type**, n (%) |  |  |  |
| NSCLC | 0 | 70 (16) | 70 (16) |
| CRC | 0 | 61 (14) | 61 (14) |
| Thyroid cancer | 5 (33) | 42 (10) | 47 (10) |
| Soft tissue sarcoma | 5 (33) | 41 (9) | 46 (10) |
| Breast cancer | 1 (7) | 43 (10) | 44 (10) |
| Pancreatic cancer | 0 | 32 (7) | 32 (7) |
| Salivary gland cancer | 0 | 32 (7) | 32 (7) |
| Other^†^ | 4 (27) | 115 (26) | 119 (26) |
| **Stage at initial diagnosis**, n (%) |  |  |  |
| I | 1 (7) | 15 (3) | 16 (4) |
| II | 1 (7) | 23 (5) | 24 (5) |
| III | 0 | 38 (9) | 38 (8) |
| I-III (NOS) | 0 | 1 (<1) | 1 (<1) |
| IV | 1 (7) | 203 (47) | 204 (45) |
| Unknown/Missing | 12 (80) | 156 (36) | 168 (37) |
| **ECOG PS at advanced/metastatic diagnosis**, n (%) |  |  |  |
| 0 | 0 | 30 (7) | 30 (7) |
| 1 | 0 | 81 (19) | 81 (18) |
| 2 | 0 | 33 (8) | 33 (7) |
| 3 | 0 | 2 (<1) | 2 (<1) |
| Missing | 15 (100) | 290 (67) | 305 (68) |
| **Metastasis status at advanced/metastatic diagnosis**, n (%) |  |  |  |
| No | 10 (67) | 229 (53) | 239 (53) |
| Yes | 5 (33) | 207 (48) | 212 (47) |
| ***NTRK* gene fusion test type**, n (%) |  |  |  |
| NGS | 11 (73) | 352 (81) | 363 (81) |
| FISH | 0 | 16 (4) | 16 (4) |
| IHC following by confirmatory NGS test | 0 | 9 (2) | 9 (2) |
| Missing/unknown | 4 (27) | 59 (14) | 63 (14) |
| **Days from advanced/metastatic diagnosis to first *NTRK* gene fusion test date** |  |  |  |
| Median (IQR) | 527 (-7–1947) | 24 (0–434) | 25 (0–459) |
| ***NTRK* gene fusion**, n (%) |  |  |  |
| *NTRK1* | 4 (27) | 222 (51) | 226 (50) |
| *NTRK3* | 7 (47) | 145 (33) | 152 (34) |
| *NTRK2* | 0 | 55 (13) | 55 (12) |
| *NTRK1, NTRK2* | 0 | 3 (<1) | 3 (<1) |
| Missing | 4 (27) | 11 (3) | 15 (3) |
| **Received radiation prior to advanced/metastatic disease**, n (%) |  |  |  |
| No | 1 (7) | 155 (36) | 156 (35) |
| Yes | 0 | 15 (3) | 15 (3) |
| Missing | 14 (93) | 266 (61) | 280 (62) |
| **Received surgery prior to advanced/metastatic disease**, n (%) |  |  |  |
| No | 1 (7) | 129 (30) | 130 (29) |
| Yes | 0 | 43 (10) | 43 (10) |
| Missing | 14 (93) | 264 (61) | 278 (62) |

^†^Other tumor types included appendix, bladder, brain, cholangiocarcinoma, esophageal, gallbladder, gastric, head and neck, kidney, liver, lung, melanoma, neuroendocrine, prostate, uterine, and cancer of unknown primary, together with GIST and unspecified sarcoma^.^ CRC, colorectal cancer; ECOG PS, Eastern Cooperative Oncology Group performance status; FISH, fluorescence in situ hybridization; GIST, gastrointestinal stromal tumor; IHC, immunohistochemistry; IQR, interquartile range; NGS, next generation sequencing; NOS, not otherwise specified; NSCLC, non-small cell lung cancer; *NTRK*, neurotrophic tyrosine receptor kinase.

**Supplementary Table 4. Frequency of *NTRK* gene fusion and fusion partners in adult patients with advanced/metastatic cancers**

| ***NTRK* gene fusion partner,  n (%)** | ***NTRK1* *n =* 226** | ***NTRK1*, *NTRK2* *n =* 3** | ***NTRK2* *n =* 55** | ***NTRK3* *n =* 152** | **Total *N =* 436** |
| --- | --- | --- | --- | --- | --- |
| *ACOI* | 1 (0.4) | 0 | 1 (1.8) | 0 | 2 (0.5) |
| *AFAP1* | 2 (0.9) | 0 | 4 (7.3) | 2 (1.3) | 8 (1.8) |
| *AGBL1* | 0 | 0 | 0 | 2 (1.3) | 2 (0.5) |
| *AMMECR1* | 0 | 0 | 0 | 1 (0.7) | 1 (0.2) |
| *APOL3* | 0 | 0 | 1 (1.8) | 0 | 1 (0.2) |
| *ARHGEF2* | 2 (0.9) | 0 | 0 | 0 | 2 (0.5) |
| *ATM* | 1 (0.4) | 0 | 0 | 0 | 1 (0.2) |
| *ATP1A2* | 1 (0.4) | 0 | 0 | 0 | 1 (0.2) |
| *BCAN* | 1 (0.4) | 0 | 0 | 0 | 1 (0.2) |
| *BCR* | 0 | 0 | 1 (1.8) | 0 | 1 (0.2) |
| *BTBD1* | 0 | 0 | 0 | 2 (1.3) | 2 (0.5) |
| *C5orf43* | 1 (0.4) | 0 | 0 | 0 | 1 (0.2) |
| *CADM3* | 1 (0.4) | 0 | 0 | 0 | 1 (0.2) |
| *CCBL1* | 0 | 0 | 1 (1.8) | 0 | 1 (0.2) |
| *CD247* | 1 (0.4) | 0 | 0 | 0 | 1 (0.2) |
| *CD74* | 2 (0.9) | 0 | 0 | 2 (1.3) | 4 (0.9) |
| *CEMIP* | 0 | 0 | 0 | 1 (0.7) | 1 (0.2) |
| *CHST11* | 0 | 0 | 0 | 1 (0.7) | 1 (0.2) |
| *CLCNKB* | 1 (0.4) | 0 | 0 | 0 | 1 (0.2) |
| *CTRC* | 1 (0.4) | 0 | 0 | 0 | 1 (0.2) |
| *CTSK* | 1 (0.4) | 0 | 0 | 0 | 1 (0.2) |
| *DAB2IP* | 0 | 0 | 1 (1.8) | 0 | 1 (0.2) |
| *DDR2* | 1 (0.4) | 0 | 0 | 0 | 1 (0.2) |
| *DOT1L* | 0 | 0 | 0 | 1 (0.7) | 1 (0.2) |
| *EFCAB11* | 0 | 0 | 1 (1.8) | 0 | 1 (0.2) |
| *EFNA3* | 1 (0.4) | 0 | 0 | 0 | 1 (0.2) |
| *EML4* | 1 (0.4) | 0 | 0 | 2 (1.3) | 3 (0.7) |
| *EPS15* | 2 (0.9) | 0 | 0 | 0 | 2 (0.5) |
| *ETV6* | 5 (2.2) | 1 (33.3) | 3 (5.5) | 76 (50.0) | 85 (19.5) |
| *FGD3* | 0 | 0 | 2 (3.6) | 0 | 2 (0.5) |
| *FMO1* | 1 (0.4) | 0 | 0 | 0 | 1 (0.2) |
| *FOXJ2* | 0 | 0 | 0 | 1 (0.7) | 1 (0.2) |
| *FRMD3* | 0 | 0 | 1 (1.8) | 0 | 1 (0.2) |
| *GKAP1* | 0 | 0 | 2 (3.6) | 0 | 2 (0.5) |
| *GRIPAP1* | 1 (0.4) | 0 | 0 | 0 | 1 (0.2) |
| *HAUS6* | 0 | 0 | 1 (1.8) | 0 | 1 (0.2) |
| *HDGF,TPR* | 1 (0.4) | 0 | 0 | 0 | 1 (0.2) |
| *IFT140* | 0 | 0 | 0 | 1 (0.7) | 1 (0.2) |
| *INTERGENIC* | 8 (3.5) | 0 | 1 (1.8) | 4 (2.6) | 13 (3.0) |
| *INTERGENIC,RP11* | 1 (0.4) | 0 | 0 | 0 | 1 (0.2) |
| *INTERGENICT,TJAP1* | 1 (0.4) | 0 | 0 | 0 | 1 (0.2) |
| *INTRAGENIC*^†^ | 24 (10.6) | 0 | 11 (20.0) | 9 (5.9) | 44 (10.1) |
| *IQGAP3* | 1 (0.4) | 0 | 0 | 0 | 1 (0.2) |
| *Irf2bp2* | 1 (0.4) | 0 | 0 | 0 | 1 (0.2) |
| *Kank1* | 0 | 0 | 1 (1.8) | 0 | 1 (0.2) |
| *Klh25* | 0 | 0 | 0 | 1 (0.7) | 1 (0.2) |
| *LMNA* | 30 (13.3) | 0 | 1 (1.8) | 3 (2.0) | 34 (7.8) |
| *MEF2D* | 1 (0.4) | 0 | 0 | 0 | 1 (0.2) |
| *MPRIP* | 1 (0.4) | 0 | 0 | 0 | 1 (0.2) |
| *MTM1* | 0 | 0 | 1 (1.8) | 0 | 1 (0.2) |
| *MYST3* | 0 | 0 | 0 | 1 (0.7) | 1 (0.2) |
| *NES* | 1 (0.4) | 0 | 0 | 0 | 1 (0.2) |
| *NFASC* | 1 (0.4) | 0 | 0 | 0 | 1 (0.2) |
| *NOS1AP* | 1 (0.4) | 0 | 0 | 0 | 1 (0.2) |
| *P2RY8* | 1 (0.4) | 0 | 0 | 0 | 1 (0.2) |
| *PAN3* | 3 (1.3) | 0 | 1 (1.8) | 1 (0.7) | 5 (1.1) |
| *PBX1* | 1 (0.4) | 0 | 0 | 0 | 1 (0.2) |
| *PDE3B* | 0 | 0 | 0 | 1 (0.7) | 1 (0.2) |
| *PDIA3* | 1 (0.4) | 0 | 0 | 0 | 1 (0.2) |
| *PEAK1* | 0 | 0 | 0 | 1 (0.7) | 1 (0.2) |
| *PEAR1* | 4 (1.8) | 0 | 0 | 0 | 4 (0.9) |
| *PKIG* | 0 | 0 | 0 | 1 (0.7) | 1 (0.2) |
| *PLA2G4A* | 1 (0.4) | 0 | 0 | 0 | 1 (0.2) |
| *PLEKHA6* | 1 (0.4) | 0 | 0 | 0 | 1 (0.2) |
| *PPL* | 5 (2.2) | 0 | 1 (1.8) | 0 | 6 (1.4) |
| *PRCC* | 1 (0.4) | 0 | 0 | 0 | 1 (0.2) |
| *PREX1* | 0 | 0 | 0 | 1 (0.7) | 1 (0.2) |
| *PTPRG* | 0 | 0 | 1 (1.8) | 0 | 1 (0.2) |
| *RAB25* | 1 (0.4) | 0 | 0 | 0 | 1 (0.2) |
| *RABGAP1L* | 2 (0.9) | 0 | 0 | 0 | 2 (0. 5) |
| *RBPMS* | 0 | 0 | 0 | 3 (2.0) | 3 (0.7) |
| *RCSD1* | 1 (0.4) | 0 | 0 | 0 | 1 (0.2) |
| *SCP2* | 1 (0.4) | 0 | 0 | 0 | 1 (0.2) |
| *SCYL3* | 1 (0.4) | 0 | 0 | 0 | 1 (0.2) |
| *SEMA4A* | 1 (0.4) | 0 | 0 | 0 | 1 (0.2) |
| *SFPQ* | 1 (0.4) | 0 | 0 | 0 | 1 (0.2) |
| *SH2D2A* | 1 (0.4) | 0 | 0 | 0 | 1 (0.2) |
| *SLC4A10* | 0 | 0 | 1 (1.8) | 0 | 1 (0.2) |
| *SMAD3* | 0 | 0 | 0 | 1 (0.7) | 1 (0.2) |
| *SND1* | 1 (0.4) | 0 | 0 | 0 | 1 (0.2) |
| *SNRPE* | 1 (0.4) | 0 | 0 | 0 | 1 (0.2) |
| *SOX6* | 0 | 0 | 1 (1.8) | 0 | 1 (0.2) |
| *SPG11* | 0 | 0 | 0 | 1 (0.7) | 1 (0.2) |
| *SPIN4* | 0 | 0 | 0 | 1 (0.7) | 1 (0.2) |
| *SQSTM1* | 7 (3.1) | 0 | 2 (3.6) | 2 (1.3) | 11 (2.5) |
| *STMN4* | 0 | 0 | 0 | 1 (0.7) | 1 (0.2) |
| *STRN* | 0 | 0 | 1 (1.8) | 0 | 1 (0.2) |
| *STX6* | 1 (0.4) | 0 | 0 | 0 | 1 (0.2) |
| *TENM4* | 1 (0.4) | 0 | 0 | 0 | 1 (0.2) |
| *TFG* | 1 (0.4) | 0 | 0 | 1 (0.7) | 2 (0.5) |
| *TP53* | 1 (0.4) | 0 | 0 | 0 | 1 (0.2) |
| *TPCN2* | 0 | 0 | 0 | 1 (0.7) | 1 (0.2) |
| *TPM1* | 1 (0.4) | 0 | 0 | 0 | 1 (0.2) |
| *TPM3* | 39 (17.3) | 0 | 5 (9.1) | 1 (0.7) | 45 (10.3) |
| *TPR* | 17 (7.5) | 0 | 0 | 0 | 17 (3.9) |
| *TRAF2* | 0 | 0 | 2 (3.6) | 0 | 2 (0.5) |
| *TRIM24* | 3 (1.3) | 0 | 1 (1.8) | 2 (1.3) | 6 (1.4) |
| *TRIM63* | 2 (0.9) | 0 | 0 | 0 | 2 (0.5) |
| *TUBGCP5* | 0 | 0 | 0 | 1 (0.7) | 1 (0.2) |
| *WASF3* | 0 | 0 | 0 | 1 (0.7) | 1 (0.2) |
| *ZBTB1* | 1 (0.4) | 0 | 0 | 0 | 1 (0.2) |
| *ZBTB7B* | 1 (0.4) | 0 | 0 | 0 | 1 (0.2) |
| *ZMAT4* | 0 | 0 | 0 | 1 (0.7) | 1 (0.2) |
| *ZNF710* | 0 | 0 | 0 | 1 (0.7) | 1 (0.2) |
| Missing/unknown | 22 (9.7) | 2 (66.7) | 5 (9.1) | 19 (12.5) | 48 (11.0) |

^†^This category reflects the source-reported annotation rather than a specific external partner gene; functional relevance could not be determined from the available data.

**Supplementary Table 5. rwRR for first LOT by tumor type in patients with advanced/metastatic cancers**

|  | **NSCLC (*n =* 64)** | **CRC  (*n =* 56)** | **Thyroid cancer  (*n =* 35)** | **Soft tissue sarcoma (*n =* 42)** | **Breast cancer (*n =* 43)** | **Salivary gland cancer (*n =* 32)** | **Pancreatic cancer  (*n =* 30)** | **Other  (*N =* 113)** |
| --- | --- | --- | --- | --- | --- | --- | --- | --- |
| **Radiologist assessment** | | | | | | | | |
| **Best overall response**, n (%) |  |  |  |  |  |  |  |  |
| Complete or partial response | 24 (38) | 13 (23) | 20 (57) | 6 (14) | 8 (19) | 10 (31) | 8 (27) | 27 (24) |
| Stable disease | 5 (8) | 2 (4) | 4 (11) | 5 (12) | 1 (2) | 3 (9) | 3 (10) | 14 (12) |
| Progressive disease | 3 (5) | 5 (9) | 2 (6) | 6 (14) | 3 (7) | 5 (16) | 5 (17) | 18 (16) |
| Missing/ undetermined | 32 (50) | 36 (64) | 9 (26) | 25 (60) | 31 (72) | 14 (44) | 14 (47) | 54 (48) |
| **ORR,** % (95% CI) |  |  |  |  |  |  |  |  |
| All patients^†^ | 38 (26–51) | 23 (13–36) | 57 (39–74) | 14 (5–29) | 19 (8–33) | 31 (16–50) | 27 (12–46) | 24 (16–33) |
| Evaluable patients^‡^ | 75 (57–89) | 65 (41–85) | 77 (56–91) | 35 (14–62) | 67 (35–90) | 56 (31–79) | 50 (25–75) | 46 (33–59) |
| **Oncologist assessment** | | | | | | | | |
| **Best overall response**, n (%) |  |  |  |  |  |  |  |  |
| Complete or partial response | 25 (39) | 13 (23) | 21 (60) | 6 (14) | 8 (19) | 9 (28) | 6 (20) | 28 (25) |
| Stable disease | 8 (13) | 2 (4) | 3 (9) | 5 (12) | 2 (5) | 3 (9) | 5 (17) | 14 (12) |
| Progressive disease | 2 (3) | 6 (11) | 1 (3) | 6 (14) | 1 (2) | 7 (22) | 4 (13) | 13 (12) |
| Missing/ undetermined | 29 (45) | 35 (63) | 10 (29) | 25 (60) | 32 (74) | 13 (41) | 15 (50) | 58 (51) |
| **ORR**, % (95% CI) |  |  |  |  |  |  |  |  |
| All patients^†^ | 39 (27–52) | 23 (13–36) | 60 (42–76) | 14 (5–29) | 19 (8–33) | 28 (14–47) | 20 (8–39) | 25 (17–34) |
| Evaluable patients^‡^ | 71 (54–85) | 62 (38–82) | 84 (64–96) | 35 (14–62) | 73 (39–94) | 47 (24–71) | 40 (16–68) | 51 (37–65) |

^†^Defined as the proportion of all patients with a best overall response of complete response or partial response during the treatment period. ^‡^Defined as the proportion of evaluable patients (i.e., missing/undetermined excluded) with a best overall response of complete response or partial response during the treatment period. CI, confidence interval; CRC, colorectal cancer; LOT, line of therapy; NSCLC, non-small cell lung cancer; ORR, overall response rate; rwRR, real-world response rate.

**Supplementary Table 6. Baseline demographics and clinical characteristics in adult and pediatric patients with an initial diagnosis of cancer by tumor type**

|  | **NSCLC (*n =* 72)** | **CRC (*n =* 68)** | **Thyroid cancer (*n =* 63)** | **Soft tissue sarcoma (*n =* 56)** | **Breast cancer (*n =* 47)** | **Salivary gland cancer (*n =* 36)** | **Pancreatic cancer (*n =* 32)** | **Other (*N =* 138)^†^** | **Total (*N =* 512)** |
| --- | --- | --- | --- | --- | --- | --- | --- | --- | --- |
| **Sex**, n (%) |  |  |  |  |  |  |  |  |  |
| Female | 32 (44) | 44 (65) | 42 (67) | 30 (54) | 45 (96) | 10 (28) | 13 (41) | 58 (42) | 274 (54) |
| Male | 40 (56) | 24 (35) | 21 (33) | 25 (45) | 2 (4) | 26 (72) | 19 (59) | 80 (58) | 237 (46) |
| Missing | 0 | 0 | 0 | 1 (2) | 0 | 0 | 0 | 0 | 1 (<1) |
| **Age at initial diagnosis,** years |  |  |  |  |  |  |  |  |  |
| Median (range) | 63 (22–84) | 61 (45–92) | 42 (7–79) | 52 (0–83) | 50 (9–79) | 57 (23–76) | 62 (39–85) | 60 (0–83) | 58 (0–92) |
| **Age group at initial diagnosis**,  n (%) |  |  |  |  |  |  |  |  |  |
| Age ≥18 years | 72 (100) | 68 (100) | 47 (75) | 49 (88) | 46 (98) | 36 (100) | 32 (100) | 130 (94) | 480 (94) |
| Age <18 years | 0 | 0 | 16 (25) | 7 (13) | 1 (2) | 0 | 0 | 8 (6) | 32 (6) |
| **Race**, n (%) |  |  |  |  |  |  |  |  |  |
| White | 60 (83) | 45 (66) | 46 (73) | 40 (71) | 34 (72) | 29 (81) | 27 (84) | 108 (78) | 389 (76) |
| Black | 4 (6) | 7 (10) | 4 (6) | 3 (5) | 7 (15) | 1 (3) | 3 (9) | 7 (5) | 36 (7) |
| Asian | 7 (10) | 3 (4) | 6 (10) | 5 (9) | 1 (2) | 3 (8) | 2 (6) | 7 (5) | 34 (7) |
| Other | 0 | 7 (10) | 4 (6) | 6 (11) | 2 (4) | 3 (8) | 0 | 8 (6) | 30 (6) |
| Unknown/missing | 1 (1) | 6 (9) | 3 (5) | 2 (4) | 3 (6) | 0 | 0 | 8 (6) | 23 (5) |
| **Ethnicity**, n (%) |  |  |  |  |  |  |  |  |  |
| Hispanic or Latino | 2 (3) | 5 (7) | 6 (10) | 6 (11) | 4 (9) | 3 (8) | 6 (19) | 6 (4) | 38 (7) |
| Not Hispanic or Latino | 57 (79) | 33 (49) | 48 (76) | 33 (59) | 31 (66) | 29 (81) | 20 (63) | 92 (67) | 343 (67) |
| Unknown/missing | 13 (18) | 30 (44) | 9 (14) | 17 (30) | 12 (26) | 4 (11) | 6 (19) | 40 (29) | 131 (26) |
| **Region**,  n (%) |  |  |  |  |  |  |  |  |  |
| South | 12 (17) | 3 (4) | 0 | 2 (4) | 2 (4) | 1 (3) | 7 (22) | 14 (10) | 41 (8) |
| West | 7 (10) | 3 (4) | 4 (6) | 3 (5) | 0 | 5 (14) | 3 (9) | 9 (7) | 34 (7) |
| Northeast | 3 (4) | 3 (4) | 0 | 2 (4) | 0 | 3 (8) | 2 (6) | 11 (8) | 24 (5) |
| Midwest | 3 (4) | 2 (3) | 2 (3) | 1 (2) | 1 (2) | 1 (3) | 0 | 1 (<1) | 11 (2) |
| Unknown/missing | 47 (65) | 57 (84) | 57 (91) | 48 (86) | 44 (94) | 26 (72) | 20 (63) | 103 (75) | 402 (79) |
| **Smoking status**,  n (%) |  |  |  |  |  |  |  |  |  |
| History of smoking | 37 (51) | 3 (4) | 4 (6) | 6 (11) | 1 (2) | 4 (11) | 10 (31) | 18 (13) | 83 (16) |
| No history of smoking | 22 (31) | 5 (7) | 6 (10) | 5 (9) | 2 (4) | 9 (25) | 4 (13) | 11 (8) | 64 (13) |
| Unknown/missing | 13 (18) | 60 (88) | 53 (84) | 45 (80) | 44 (94) | 23 (64) | 18 (56) | 109 (79) | 365 (71) |
| **Stage at initial diagnosis**,  n (%) |  |  |  |  |  |  |  |  |  |
| I | 4 (6) | 0 | 3 (5) | 6 (11) | 4 (9) | 2 (6) | 1 (3) | 2 (1) | 22 (4) |
| II | 1 (1) | 5 (7) | 6 (10) | 4 (7) | 4 (9) | 2 (6) | 0 | 6 (4) | 28 (6) |
| III | 4 (6) | 15 (22) | 4 (6) | 3 (5) | 7 (15) | 3 (8) | 1 (3) | 9 (7) | 46 (9) |
| I–III (NOS) | 0 | 0 | 0 | 0 | 0 | 0 | 0 | 1 (<1) | 1 (<1) |
| IV | 48 (67) | 32 (47) | 9 (14) | 16 (29) | 10 (21) | 9 (25) | 23 (72) | 58 (42) | 205 (40) |
| Unknown/missing | 15 (21) | 16 (24) | 41 (65) | 27 (48) | 22 (47) | 20 (56) | 7 (22) | 62 (45) | 210 (41) |
| **ECOG PS at initial diagnosis**,  n (%) |  |  |  |  |  |  |  |  |  |
| 0 | 0 | 2 (3) | 4 (6) | 3 (5) | 0 | 1 (3) | 0 | 0 | 10 (2) |
| 1 | 3 (4) | 1 (2) | 0 | 1 (2) | 0 | 1 (3) | 0 | 0 | 6 (1) |
| 2 | 1 (1) | 0 | 0 | 0 | 0 | 1 (3) | 0 | 0 | 2 (<1) |
| Missing | 68 (94) | 65 (96) | 59 (94) | 52 (93) | 47 (100) | 33 (92) | 32 (100) | 138 (100) | 494 (97) |
| **Metastasis status at initial diagnosis**,  n (%) |  |  |  |  |  |  |  |  |  |
| No | 60 (83) | 49 (72) | 54 (86) | 47 (84) | 39 (83) | 36 (100) | 21 (66) | 116 (84) | 422 (82) |
| Yes | 12 (17) | 19 (28) | 9 (14) | 9 (16) | 8 (17) | 0 | 11 (34) | 22 (16) | 90 (18) |
| ***NTRK* gene fusion test type**, n (%) |  |  |  |  |  |  |  |  |  |
| NGS | 59 (82) | 62 (91) | 43 (68) | 43 (77) | 43 (92) | 27 (75) | 23 (72) | 114 (83) | 414 (81) |
| FISH | 5 (7) | 1 (2) | 0 | 0 | 2 (4) | 0 | 3 (9) | 5 (4) | 16 (3) |
| IHC followed by confirmatory NGS | 1 (1) | 1 (2) | 0 | 2 (4) | 0 | 1 (3) | 1 (3) | 3 (2) | 9 (2) |
| Missing/ unknown | 7 (10) | 4 (6) | 20 (32) | 11 (20) | 2 (4) | 8 (22) | 5 (16) | 16 (12) | 73 (14) |
| **Days from initial index date to first *NTRK* gene fusion test date** |  |  |  |  |  |  |  |  |  |
| Median (IQR) | 34  (1–370) | 58  (14–478) | 1106  (59–4383) | 69  (7–745) | 1102  (91–2797) | 513  (42–1914) | 19  (6–89) | 52  (11–711) | 86  (11–876) |
| ***NTRK* gene fusion**,  n (%) |  |  |  |  |  |  |  |  |  |
| *NTRK1* | 44 (61) | 50 (74) | 21 (33) | 31 (55) | 18 (38) | 5 (14) | 12 (38) | 74 (54) | 255 (50) |
| *NTRK3* | 13 (18) | 12 (18) | 30 (48) | 18 (32) | 22 (47) | 30 (83) | 11 (34) | 38 (28) | 174 (34) |
| *NTRK2* | 12 (17) | 5 (7) | 2 (3) | 3 (5) | 5 (11) | 1 (3) | 7 (22) | 21 (15) | 56 (11) |
| *NTRK1, NTRK2* | 1 (1) | 1 (2) | 0 | 0 | 1 (2) | 0 | 0 | 0 | 3 (<1) |
| Missing | 2 (3) | 0 | 10 (16) | 4 (7) | 1 (2) | 0 | 2 (6) | 5 (4) | 24 (5) |

^†^Other tumor types included appendix, bladder, brain, cholangiocarcinoma, esophageal, gallbladder, gastric, head and neck, kidney, liver, lung, melanoma, neuroendocrine, prostate, uterine, and cancer of unknown primary, together with GIST and unspecified sarcoma. CRC, colorectal cancer; ECOG PS, Eastern Cooperative Oncology Group performance status; FISH, fluorescence in situ hybridization; ICAS, initial cancer analysis set; IHC, immunohistochemistry; IQR, interquartile range; NGS, next-generation sequencing; NOS, not otherwise specified; NSCLC, non-small cell lung cancer.

**Supplementary Table 7. Baseline and clinical characteristics in adult and pediatric patients with an initial diagnosis of cancer**

|  | **Age <18 years**  ***N =* 32** | **Age ≥18 years**  ***N =* 480** | **Total**  ***N =* 512** |
| --- | --- | --- | --- |
| **Sex**, n (%) |  |  |  |
| Female | 23 (72) | 251 (52) | 274 (54) |
| Male | 8 (25) | 229 (48) | 237 (46) |
| Missing | 1 (3) | 0 | 1 (<1) |
| **Age at initial diagnosis,** years |  |  |  |
| Median (range) | 12 (0–18) | 59 (18–92) | 58 (0–92) |
| **Race**, n (%) |  |  |  |
| White | 15 (47) | 374 (78) | 389 (76) |
| Black | 4 (13) | 32 (7) | 36 (7) |
| Asian | 1 (3) | 33 (7) | 34 (7) |
| Other | 8 (25) | 22 (5) | 30 (6) |
| Unknown/missing | 4 (13) | 19 (4) | 23 (5) |
| **Ethnicity**, n (%) |  |  |  |
| Hispanic or Latino | 8 (25) | 30 (6) | 38 (7) |
| Not Hispanic or Latino | 18 (56) | 325 (68) | 343 (67) |
| Unknown/missing | 6 (19) | 125 (26) | 131 (26) |
| **Region**, n (%) |  |  |  |
| South | 0 | 41(9) | 41 (8) |
| West | 0 | 34 (7) | 34 (7) |
| Northeast | 0 | 24 (5) | 24 (5) |
| Midwest | 0 | 11 (2) | 11 (2) |
| Unknown/missing | 32 (100) | 370 (77) | 402 (79) |
| **Smoking status**, n (%) |  |  |  |
| History of smoking | 0 | 83 (17) | 83 (16) |
| No history of smoking | 0 | 64 (13) | 64 (13) |
| Unknown/missing | 32 (100) | 333 (69) | 365 (71) |
| **Tumor type**, n (%) |  |  |  |
| NSCLC | 0 | 72 (15) | 72 (14) |
| CRC | 0 | 68 (14) | 68 (13) |
| Thyroid cancer | 16 (50) | 47 (10) | 63 (12) |
| Soft tissue sarcoma | 7 (22) | 49 (10) | 56 (11) |
| Breast cancer | 1 (3) | 46 (10) | 47 (9) |
| Salivary gland cancer | 0 | 36 (8) | 36 (7) |
| Pancreatic cancer | 0 | 32 (7) | 32 (6) |
| Other^†^ | 8 (25) | 130 (27) | 138 (27) |
| **Stage at initial diagnosis**, n (%) |  |  |  |
| I | 2 (6) | 20 (4) | 22 (4) |
| II | 1 (3) | 27 (6) | 28 (6) |
| III | 0 | 46 (10) | 46 (9) |
| I–III (NOS) | 0 | 1 (<1) | 1 (<1) |
| IV | 1 (3) | 204 (43) | 205 (40) |
| Unknown/missing | 28 (88) | 182 (38) | 210 (41) |
| **ECOG PS at initial diagnosis**, n (%) |  |  |  |
| 0 | 2 (6) | 8 (2) | 10 (2) |
| 1 | 0 | 6 (1) | 6 (1) |
| 2 | 0 | 2 (<1) | 2 (<1) |
| Missing | 30 (94) | 464 (97) | 494 (97) |
| **Metastasis status at initial diagnosis**, n (%) |  |  |  |
| No | 31 (97) | 391 (82) | 422 (82) |
| Yes | 1 (3) | 89 (19) | 90 (18) |
| ***NTRK* gene fusion test type**, n(%) |  |  |  |
| NGS | 21 (66) | 393 (82) | 414 (81) |
| FISH | 0 | 16 (3) | 16 (3) |
| IHC (followed by confirmatory NGS) | 0 | 9 (2) | 9 (2) |
| Missing/Unknown | 11 (34) | 62 (13) | 73 (14) |
| **Days from initial diagnosis to first *NTRK* gene fusion test date** |  |  |  |
| Median (IQR) | 530 (12–2004) | 82 (11–835) | 86 (11–876) |
| ***NTRK* gene fusion**, n (%) |  |  |  |
| *NTRK1* | 9 (28) | 246 (51) | 255 (50) |
| *NTRK3* | 12 (38) | 162 (34) | 174 (34) |
| *NTRK2* | 0 | 56 (12) | 56 (11) |
| *NTRK1, NTRK2* | 0 | 3 (0.6) | 3 (<1) |
| Missing | 11 (34) | 13 (3) | 24 (5) |

^†^Other tumor types included appendix, bladder, brain, cholangiocarcinoma, esophageal, gallbladder, gastric, head and neck, kidney, liver, lung, melanoma, neuroendocrine, prostate, uterine, and cancer of unknown primary, together with GIST and unspecified sarcoma. CRC, colorectal cancer; ECOG PS, Eastern Cooperative Oncology Group performance status; FISH, fluorescence in situ hybridization; GIST, gastrointestinal stromal tumor; IHC, immunohistochemistry; IQR, interquartile range; NGS, next generation sequencing; NOS, not otherwise specified; NSCLC, non-small cell lung cancer; *NTRK*, neurotrophic tyrosine receptor kinase.

**Supplementary Table 8. Frequency of *NTRK* gene fusion and fusion partners in patients with an initial diagnosis of cancer**

| ***NTRK* gene fusion partner,  n (%)** | ***NTRK1* *n =* 255** | ***NTRK1*, *NTRK2* *n =* 3** | ***NTRK2* *n =* 56** | ***NTRK3* *n =* 174** | **Total *N =* 488** |
| --- | --- | --- | --- | --- | --- |
| *ACOI* | 1 (0.4) | 0 | 1 (1.8) | 0 | 2 (0.4) |
| *AFAP1* | 2 (0.8) | 0 | 4 (7.1) | 2 (1.1) | 8 (1.6) |
| *AGBL1* | 0 | 0 | 0 | 2 (1.1) | 2 (0.4) |
| *ALLC* | 0 | 0 | 0 | 1 (0.6) | 1 (0.2) |
| *AMMECR1* | 0 | 0 | 0 | 1 (0.6) | 1 (0.2) |
| *APOL3* | 0 | 0 | 1 (1.8) | 0 | 1 (0.2) |
| *ARHGEF2* | 3 (1.2) | 0 | 0 | 0 | 3 (0.6) |
| *ATM* | 1 (0.4) | 0 | 0 | 0 | 1 (0.2) |
| *ATP1A2* | 1 (0.4) | 0 | 0 | 0 | 1 (0.2) |
| *BCAN* | 1 (0.4) | 0 | 0 | 0 | 1 (0.2) |
| *BCR* | 0 | 0 | 1 (1.8) | 0 | 1 (0.2) |
| *BTBD1* | 0 | 0 | 0 | 2 (1.1) | 2 (0.4) |
| *C5orf42* | 1 (0.4) | 0 | 0 | 0 | 1 (0.2) |
| *CADM3* | 1 (0.4) | 0 | 0 | 0 | 1 (0.2) |
| *CCBL1* | 0 | 0 | 1 (1.8) | 0 | 1 (0.2) |
| *CD247* | 1 (0.4) | 0 | 0 | 0 | 1 (0.2) |
| *CD74* | 2 (0.8) | 0 | 0 | 2 (1.1) | 4 (0.8) |
| *CEMIP* | 0 | 0 | 0 | 1 (0.6) | 1 (0.2) |
| *CHST11* | 0 | 0 | 0 | 1 (0.6) | 1 (0.2) |
| *CLCNKB* | 1 (0.4) | 0 | 0 | 0 | 1 (0.2) |
| *CTRC* | 1 (0.4) | 0 | 0 | 0 | 1 (0.2) |
| *CTSK* | 1 (0.4) | 0 | 0 | 0 | 1 (0.2) |
| *DAB2IP* | 0 | 0 | 1 (1.8) | 0 | 1 (0.2) |
| *DDR2* | 1 (0.4) | 0 | 0 | 0 | 1 (0.2) |
| *DOT1L* | 0 | 0 | 0 | 1 (0.6) | 1 (0.2) |
| *DUSP10,TPM3* | 1 (0.4) | 0 | 0 | 0 | 1 (0.2) |
| *EFCAB11* | 0 | 0 | 1 (1.8) | 0 | 1 (0.2) |
| *EFNA3* | 1 (0.4) | 0 | 0 | 0 | 1 (0.2) |
| *EML4* | 1 (0.4) | 0 | 0 | 6 (3.4) | 7 (1.4) |
| *EPS15* | 3 (1.2) | 0 | 0 | 0 | 3 (0.6) |
| *ETV3,TPR* | 1 (0.4) | 0 | 0 | 0 | 1 (0.2) |
| *ETV6* | 5 (2.0) | 1 (33.3) | 3 (5.4) | 90 (51.7) | 99 (20.3) |
| *FAM19A2* | 1 (0.4) | 0 | 0 | 0 | 1 (0.2) |
| *FGD3* | 0 | 0 | 2 (3.6) | 0 | 2 (0.4) |
| *FMO1* | 1 (0.4) | 0 | 0 | 0 | 1 (0.2) |
| *FOXJ2* | 0 | 0 | 0 | 1 (0.6) | 1 (0.2) |
| *FRMD3* | 0 | 0 | 1 (1.8) | 0 | 1 (0.2) |
| *GKAP1* | 0 | 0 | 2 (3.6) | 0 | 2 (0.4) |
| *GNAQ* | 0 | 0 | 1 (1.8) | 0 | 1 (0.2) |
| *GRIPAP1* | 1 (0.4) | 0 | 0 | 0 | 1 (0.2) |
| *HAUS6* | 0 | 0 | 1 (1.8) | 0 | 1 (0.2) |
| *HDGF,TPR* | 1 (0.4) | 0 | 0 | 0 | 1 (0.2) |
| *IFT140* | 0 | 0 | 0 | 1 (0.6) | 1 (0.2) |
| *ILDR2* | 1 (0.4) | 0 | 0 | 0 | 1 (0.2) |
| *INTERGENIC* | 9 (3.5) | 0 | 1 (1.8) | 4 (2.3) | 14 (2.9) |
| *INTERGENIC,RP11* | 1 (0.4) | 0 | 0 | 0 | 1 (0.2) |
| *INTERGENICT,TJAP1* | 1 (0.4) | 0 | 0 | 0 | 1 (0.2) |
| *INTERGENIC,TPR* | 1 (0.4) | 0 | 0 | 0 | 1 (0.2) |
| *INTRAGENIC*^†^ | 28 (11.0) | 0 | 11 (19.6) | 9 (5.2) | 48 (9.8) |
| *IQGAP3* | 1 (0.4) | 0 | 0 | 0 | 1 (0.2) |
| *Irf2bp2* | 1 (0.4) | 0 | 0 | 0 | 1 (0.2) |
| *Kank1* | 0 | 0 | 1 (1.8) | 0 | 1 (0.2) |
| *Klh25* | 0 | 0 | 0 | 1 (0.6) | 1 (0.2) |
| *LMNA* | 32 (12.5) | 0 | 1 (1.8) | 3 (1.7) | 36 (7.4) |
| *MEF2D* | 2 (0.8) | 0 | 0 | 0 | 2 (0.4) |
| *MEX3A* | 1 (0.4) | 0 | 0 | 0 | 1 (0.2) |
| *MPRIP* | 1 (0.4) | 0 | 0 | 0 | 1 (0.2) |
| *MTA1* | 1 (0.4) | 0 | 0 | 0 | 1 (0.2) |
| *MTM1* | 0 | 0 | 1 (1.8) | 0 | 1 (0.2) |
| *MYST3* | 0 | 0 | 0 | 1 (0.6) | 1 (0.2) |
| *NES* | 1 (0.4) | 0 | 0 | 0 | 1 (0.2) |
| *NFASC* | 1 (0.4) | 0 | 0 | 0 | 1 (0.2) |
| *NOS1AP* | 1 (0.4) | 0 | 0 | 0 | 1 (0.2) |
| *P2RY8* | 1 (0.4) | 0 | 0 | 0 | 1 (0.2) |
| *PAN3* | 3 (1.2) | 0 | 1 (1.8) | 1 (0.6) | 5 (1.0) |
| *PBX1* | 1 (0.4) | 0 | 0 | 0 | 1 (0.2) |
| *PDE3B* | 0 | 0 | 0 | 1 (0.6) | 1 (0.2) |
| *PDE8A* | 0 | 0 | 0 | 1 (0.6) | 1 (0.2) |
| *PDIA3* | 1 (0.4) | 0 | 0 | 0 | 1 (0.2) |
| *PEAK1* | 0 | 0 | 0 | 1 (0.6) | 1 (0.2) |
| *PEAR1* | 4 (1.6) | 0 | 0 | 0 | 4 (0.8) |
| *PKIG* | 0 | 0 | 0 | 1 (0.6) | 1 (0.2) |
| *PLA2G4A* | 1 (0.4) | 0 | 0 | 0 | 1 (0.2) |
| *PLEKHA6* | 2 (0.8) | 0 | 0 | 0 | 2 (0.4) |
| *PPL* | 5 (2.0) | 0 | 1 (1.8) | 0 | 6 (1.2) |
| *PRCC* | 1 (0.4) | 0 | 0 | 0 | 1 (0.2) |
| *PREX1* | 0 | 0 | 0 | 1 (0.6) | 1 (0.2) |
| *PTPRG* | 0 | 0 | 1 (1.8) | 0 | 1 (0.2) |
| *RAB25* | 1 (0.4) | 0 | 0 | 0 | 1 (0.2) |
| *RABGAP1L* | 2 (0.8) | 0 | 0 | 0 | 2 (0.4) |
| *RBPMS* | 0 | 0 | 0 | 3 (1.7) | 3 (0.6) |
| *RCSD1* | 1 (0.4) | 0 | 0 | 0 | 1 (0.2) |
| *SCP2* | 1 (0.4) | 0 | 0 | 0 | 1 (0.2) |
| *SCYL3* | 1 (0.4) | 0 | 0 | 0 | 1 (0.2) |
| *SEMA4A* | 1 (0.4) | 0 | 0 | 0 | 1 (0.2) |
| *SFPQ* | 1 (0.4) | 0 | 0 | 0 | 1 (0.2) |
| *SH2D2A* | 1 (0.4) | 0 | 0 | 0 | 1 (0.2) |
| *SLC4A10* | 0 | 0 | 1 (1.8) | 0 | 1 (0.2) |
| *SMAD3* | 0 | 0 | 0 | 1 (0.6) | 1 (0.2) |
| *SND1* | 1 (0.4) | 0 | 0 | 0 | 1 (0.2) |
| *SNRPE* | 1 (0.4) | 0 | 0 | 0 | 1 (0.2) |
| *SOX13* | 1 (0.4) | 0 | 0 | 0 | 1 (0.2) |
| *SOX6* | 0 | 0 | 1 (1.8) | 0 | 1 (0.2) |
| *SPG11* | 0 | 0 | 0 | 1 (0.6) | 1 (0.2) |
| *SPIN4* | 0 | 0 | 0 | 1 (0.6) | 1 (0.2) |
| *SQSTM1* | 7 (2.7) | 0 | 2 (3.6) | 4 (2.3) | 13 (2.7) |
| *SRGAP2* | 1 (0.4) | 0 | 0 | 0 | 1 (0.2) |
| *STMN4* | 0 | 0 | 0 | 1 (0.6) | 1 (0.2) |
| *STRN* | 0 | 0 | 1 (1.8) | 0 | 1 (0.2) |
| *STX6* | 1 (0.4) | 0 | 0 | 0 | 1 (0.2) |
| *TENM4* | 1 (0.4) | 0 | 0 | 0 | 1 (0.2) |
| *TFG* | 1 (0.4) | 0 | 0 | 1 (0.6) | 2 (0.4) |
| *TP53* | 1 (0.4) | 0 | 0 | 0 | 1 (0.2) |
| *TPCN2* | 0 | 0 | 0 | 1 (0.6) | 1 (0.2) |
| *TPM1* | 1 (0.4) | 0 | 0 | 0 | 1 (0.2) |
| *TPM3* | 44 (17.3) | 0 | 5 (8.9) | 1 (0.6) | 50 (10.2) |
| *TPR* | 21 (8.2) | 0 | 0 | 0 | 21 (4.3) |
| *TRAF2* | 0 | 0 | 2 (3.6) | 0 | 2 (0.4) |
| *TRIM24* | 3 (1.2) | 0 | 1 (1.8) | 2 (1.1) | 6 (1.2) |
| *TRIM63* | 2 (0.8) | 0 | 0 | 0 | 2 (0.4) |
| *TUBGCP5* | 0 | 0 | 0 | 1 (0.6) | 1 (0.2) |
| *WASF3* | 0 | 0 | 0 | 1 (0.6) | 1 (0.2) |
| *ZBTB1* | 1 (0.4) | 0 | 0 | 0 | 1 (0.2) |
| *ZBTB7B* | 1 (0.4) | 0 | 0 | 0 | 1 (0.2) |
| *ZMAT4* | 0 | 0 | 0 | 1 (0.6) | 1 (0.2) |
| *ZNF710* | 0 | 0 | 0 | 1 (0.6) | 1 (0.2) |
| Missing/unknown | 22 (8.6) | 2 (66.7) | 5 (8.9) | 19 (10.9) | 48 (9.8) |

^†^This category reflects the source-reported annotation rather than a specific external partner gene; functional relevance could not be determined from the available data.

**Supplementary Figure 1. CONSORT diagram of included patients**

AACR GENIE, American Association for Cancer Research Genomics Evidence Neoplasia Information Exchange; FH-FMI CGDB, Flatiron Health-Foundation Medicine Clinico-Genomic Database; ORIEN, Oncology Research Information Exchange Network.

**Supplementary Figure 2. rwOS^†^ (A), DOT (B), TTNT (C), and rwPFS (D) by LOT number in patients with advanced/metastatic cancers**

^†^Left truncation analysis was applied in estimating rwOS in adult patients only. CI, confidence interval; DOT, duration of therapy; IQR, interquartile range; LOT, line of therapy; NE, not estimable; rwOS, real-world overall survival; rwPFS, real-world progression-free survival; TTNT, time to next treatment.

**Supplementary Figure 3. Therapy type in patients with advanced/metastatic NSCLC by line of therapy number**

**Note:** Across the full dataset, targeted (small-molecule) therapies included afatinib, alectinib, entrectinib, erlotinib, imatinib, larotrectinib, lenvatinib, palbociclib, and sorafenib, while other therapy types included combination therapies, radiopharmaceuticals, and other antineoplastics.

LOT, line of therapy; NSCLC, non-small cell lung cancer.

**Supplementary Figure 4. Therapy type in patients with advanced/metastatic CRC by line of therapy number**

**Note:** Across the full dataset, targeted (small-molecule) therapies included afatinib, alectinib, entrectinib, erlotinib, imatinib, larotrectinib, lenvatinib, palbociclib, and sorafenib, while other therapy types included combination therapies, radiopharmaceuticals, and other antineoplastics.

CRC, colorectal cancer; LOT, line of therapy.

**Supplementary Figure 5. Therapy type in patients with advanced/metastatic thyroid cancer by line of therapy number**

**Note:** Across the full dataset, targeted (small-molecule) therapies included afatinib, alectinib, entrectinib, erlotinib, imatinib, larotrectinib, lenvatinib, palbociclib, and sorafenib, while other therapy types included combination therapies, radiopharmaceuticals, and other antineoplastics.

LOT, line of therapy.

**Supplementary Figure 6. Therapy type in patients with advanced/metastatic soft tissue sarcoma by line of therapy number**

**Note:** Across the full dataset, targeted (small-molecule) therapies included afatinib, alectinib, entrectinib, erlotinib, imatinib, larotrectinib, lenvatinib, palbociclib, and sorafenib, while other therapy types included combination therapies, radiopharmaceuticals, and other antineoplastics.

LOT, line of therapy.

**Supplementary Figure 7. Therapy type in patients with advanced/metastatic breast cancer by line of therapy number**

**Note:** Across the full dataset, targeted (small-molecule) therapies included afatinib, alectinib, entrectinib, erlotinib, imatinib, larotrectinib, lenvatinib, palbociclib, and sorafenib, while other therapy types included combination therapies, radiopharmaceuticals, and other antineoplastics.

LOT, line of therapy.

**Supplementary Figure 8. Therapy type in patients with advanced/metastatic salivary gland cancer by line of therapy number**

**Note:** Across the full dataset, targeted (small-molecule) therapies included afatinib, alectinib, entrectinib, erlotinib, imatinib, larotrectinib, lenvatinib, palbociclib, and sorafenib, while other therapy types included combination therapies, radiopharmaceuticals, and other antineoplastics.

LOT, line of therapy.

**Supplementary Figure 9. Therapy type in patients with advanced/metastatic pancreatic cancer by line of therapy number**

**Note:** Across the full dataset, targeted (small-molecule) therapies included afatinib, alectinib, entrectinib, erlotinib, imatinib, larotrectinib, lenvatinib, palbociclib, and sorafenib, while other therapy types included combination therapies, radiopharmaceuticals, and other antineoplastics.

LOT, line of therapy.

**Supplementary Figure 10. Therapy type in patients with advanced/metastatic other cancers by line of therapy number**

**Note:** Across the full dataset, targeted (small-molecule) therapies included afatinib, alectinib, entrectinib, erlotinib, imatinib, larotrectinib, lenvatinib, palbociclib, and sorafenib, while other therapy types included combination therapies, radiopharmaceuticals, and other antineoplastics.

LOT, line of therapy.

**Supplementary Figure 11. Oncoprint of co-occurring mutations present in ≥10% patients with an initial diagnosis of cancer^†^**

^†^Includes data only from the AACR GENIE and FH-FMI CGDB data sources. Mutations of high and medium (i.e. known or likely) impact were included

# References

1.	Pan X, Jiao X, Zong J, et al. The complexity of finding fit-for-purpose real-world data for oncology patients with rare NTRK gene fusions and a novel solution. *ESMO Real World Data Digit Oncol*. 2025;8:100139. doi: 10.1016/j.esmorw.2025.100139.

2.	American Association of Cancer Research. AACR project GENIE: powering precision medicine. Accessed December 8, 2023. <https://www.aacr.org/professionals/research/aacr-project-genie/>.

3.	Feinberg BA, Zettler ME, Klink AJ, et al. Comparison of solid tumor treatment response observed in clinical practice with response reported in clinical trials. *JAMA Netw Open*. 2021;4(2):e2036741. doi: 10.1001/jamanetworkopen.2020.36741.

4.	Flatiron Health Inc. Flatiron and Foundation Medicine’s real-world Clinico-Genomic Database (CGDB). Accessed December 19, 2023. <https://flatiron.com/real-world-evidence/clinico-genomic-database-cgdb>.

5.	Singal G, Miller PG, Agarwala V, et al. Association of patient characteristics and tumor genomics with clinical outcomes among patients with non–small cell lung cancer using a clinicogenomic database. *J Am Med Assoc*. 2019;321(14):1391-1399. doi: 10.1001/jama.2019.3241.

6.	Frampton GM, Fichtenholtz A, Otto GA, et al. Development and validation of a clinical cancer genomic profiling test based on massively parallel DNA sequencing. *Nat Biotechnol*. 2013;31(11):1023-1031. doi: 10.1038/nbt.2696.

7.	He J, Abdel-Wahab O, Nahas MK, et al. Integrated genomic DNA/RNA profiling of hematologic malignancies in the clinical setting. *Blood*. 2016;127(24):3004-3014. doi: 10.1182/blood-2015-08-664649.

8.	Woodhouse R, Li M, Hughes J, et al. Clinical and analytical validation of FoundationOne Liquid CDx, a novel 324-Gene cfDNA-based comprehensive genomic profiling assay for cancers of solid tumor origin. *PLoS One*. 2020;15(9):e0237802. doi: 10.1371/journal.pone.0237802.

9.	Willis C, Au T, Hejazi A, et al. Clinical characteristics and treatment patterns of patients with NTRK fusion-positive solid tumors: A multisite cohort study at US academic cancer centers. *J Manag Care Spec Pharm*. 2024;30(7):672-683. doi: 10.18553/jmcp.2024.30.7.672.

10.	ClinicalTrials.gov. A Study Called VICTORIA to Learn More About How Well Larotrectinib Works in Adults With TRK Fusion-positive Cancer by Comparing Larotrectinib Data From Clinical Studies With Data of Other Treatments From Actual Practice. Accessed February 16, 2024. <https://clinicaltrials.gov/study/NCT05192642>.

11.	Forsythe A, Zhang W, Phillip Strauss U, et al. A systematic review and meta-analysis of neurotrophic tyrosine receptor kinase gene fusion frequencies in solid tumors. *Ther Adv Med Oncol*. 2020;12:1758835920975613. doi: 10.1177/1758835920975613.
